# A Neurocomputational Model of Goal-Directed Navigation in Insect-Inspired Artificial Agents

**DOI:** 10.3389/fnbot.2017.00020

**Published:** 2017-04-12

**Authors:** Dennis Goldschmidt, Poramate Manoonpong, Sakyasingha Dasgupta

**Affiliations:** ^1^Bernstein Center for Computational Neuroscience, Third Institute of Physics – Biophysics, Georg-August UniversityGöttingen, Germany; ^2^Champalimaud Neuroscience Programme, Champalimaud Centre for the UnknownLisbon, Portugal; ^3^Embodied AI and Neurorobotics Lab, Centre of BioRobotics, The Mærsk Mc-Kinney Møller Institute, University of Southern DenmarkOdense, Denmark; ^4^IBM ResearchTokyo, Japan; ^5^Riken Brain Science InstituteSaitama, Japan

**Keywords:** path integration, artificial intelligence, insect navigation, neural networks, reward-based learning

## Abstract

Despite their small size, insect brains are able to produce robust and efficient navigation in complex environments. Specifically in social insects, such as ants and bees, these navigational capabilities are guided by orientation directing vectors generated by a process called path integration. During this process, they integrate compass and odometric cues to estimate their current location as a vector, called the home vector for guiding them back home on a straight path. They further acquire and retrieve path integration-based vector memories globally to the nest or based on visual landmarks. Although existing computational models reproduced similar behaviors, a neurocomputational model of vector navigation including the acquisition of vector representations has not been described before. Here we present a model of neural mechanisms in a modular closed-loop control—enabling vector navigation in artificial agents. The model consists of a path integration mechanism, reward-modulated global learning, random search, and action selection. The path integration mechanism integrates compass and odometric cues to compute a vectorial representation of the agent's current location as neural activity patterns in circular arrays. A reward-modulated learning rule enables the acquisition of vector memories by associating the local food reward with the path integration state. A motor output is computed based on the combination of vector memories and random exploration. In simulation, we show that the neural mechanisms enable robust homing and localization, even in the presence of external sensory noise. The proposed learning rules lead to goal-directed navigation and route formation performed under realistic conditions. Consequently, we provide a novel approach for vector learning and navigation in a simulated, situated agent linking behavioral observations to their possible underlying neural substrates.

## 1. Introduction

Social insects, including ants and bees, have evolved remarkable behavioral capabilities for navigating in complex dynamic environments, which enable them to survive by finding vital locations (e.g., food sources). For example, desert ants are able to forage and find small, sparsely distributed food items in a featureless environment, and form stereotyped and efficient routes between their nest and reliable food sources (Collett, 2012; Mangan and Webb, 2012; Collett and Cardé, 2014; Cheng et al., 2014). These navigational behaviors not only rely on sensory information, mainly from visual cues, but also on internal memories acquired through learning mechanisms (Collett et al., 2013). Such learned memories have shown to be based on orientation directing vectors, which are generated by a process called path integration (PI) (Wehner, 2003).

### 1.1. Vector navigation in social insects

In PI, animals integrate angular and linear ego-motion cues over time to produce an estimate of their current location with respect to their starting point. This vector representation is called the home vector (HV) and is used by social insects to return back to the home on a straight path. Many animals have been shown to apply PI, including vertebrate (Etienne and Jeffery, 2004) and invertebrate species (Srinivasan, 2015). While PI has mainly been observed in homing behavior, it can also serve as a scaffold for spatial learning of food sources (Collett et al., 1999, 2013). Indeed, experiments have shown that desert ants are capable of forming such memories by using their path integrator (Schmid-Hempel, 1984; Collett et al., 1999). Such memory is interpreted as a so-called global vector (GV), because the vector origin is fixed to the nest (Collett et al., 1998). If the ant is forced to take a detour during a foraging trip, the deviation from the GV is compensated by comparing the GV with the current PI state (Collett et al., 1999). Another example of vector memory is the waggle dance of honeybees (De Marco and Menzel, 2005; Menzel et al., 2005), in which the distance and direction to a goal are encoded by the duration and direction of the dance, respectively. After returning from a successful foraging run, insects re-apply this vector information in subsequent foraging runs (Capaldi et al., 2000; Wolf et al., 2012; Fernandes et al., 2015).

Although PI plays a key role in navigating through environments where visual cues, such as landmarks, are abundant, it also influences navigational behaviors in cluttered environments (Bühlmann et al., 2011). If an ant follows a learned GV repeatedly, it learns the heading directions at local landmarks along the path (Collett and Collett, 2009). These heading directions are view-based from the visual panorama surrounding the ant (Graham and Cheng, 2009; Narendra et al., 2013), and vector-based with additional information about the path segment length (Collett and Collett, 2009, 2015). The latter vector memories are also termed local vectors, because their retrieval is linked to local landmarks instead of global location with respect to the nest (Collett et al., 1998). Besides spatial learning of locations and routes, searching patterns of desert ants have also shown to be influenced by PI (Bolek and Wolf, 2015; Pfeffer et al., 2015).

### 1.2. Neural substrates of social insect navigation

Neural substrates of social insect navigation have yet to be completely identified, but previous findings of neural representations of compass cues and visual sceneries may provide essential information about how PI and vector learning is achieved in neural systems (Duer et al., 2015; Plath and Barron, 2015; Seelig and Jayaraman, 2015; Weir and Dickinson, 2015). In particular, neurons in the central complex, a protocerebral neuropil in the insect brain, have shown to be involved in visually guided navigation.

The main sensory cue for PI in social insects is derived from the linear polarization of scattered sunlight (Homberg et al., 2011; Lebhardt et al., 2012; Evangelista et al., 2014). Specialized photoreceptors in the outer dorsal part of the insect eye detect certain orientations of linear polarization, which depend on the azimuthal position of the sun. A distinct neural pathway processes polarization-derived signals leading to neurons in the central complex, which encode azimuthal directions of the sun (Heinze and Homberg, 2007). In a recent study, Seelig and Jayaraman (2015) placed a fruit fly tethered on a track ball setup in a virtual environment and measured the activity of neurons in the central complex. They demonstrated that certain neurons in the ellipsoid body, which is a toroidal subset in the central complex, encode for the animal's body orientation based on visual landmarks and angular self-motion. When both visual and self-motion cues are absent, this representation is maintained through persistent activity, which is a potential neural substrate for short-term memory in insects (Dubnau and Chiang, 2013). A similar neural code of orientations has been found in the rat limbic system (Taube et al., 1990). These so-called head direction (HD) cells are derived from motor and vestibular sensory information by integrating head movements through space. Thus, neural substrates of allothetic compass cues have been found in both invertebrate and vertebrate species. These cues provide input signals for a potential PI mechanism based on the accumulation of azimuthal directions of the moving animal as previously proposed by Kubie and Fenton (2009).

### 1.3. Computational models of vector-guided navigation

Because spatial navigation is a central task of biological as well as artificial agents, many studies have focused on computational modeling of such behavioral capabilities (see Madl et al., 2015 for review). Computational modeling has been successful in exploring the link between neural structures and their behavioral function, including learning (Bienenstock et al., 1982; Oja, 1982), perception (Salinas and Abbott, 1995; Olshausen and Field, 1997), and motor control (Todorov and Jordan, 2002). It allows for hypotheses about the underlying mechanisms to be defined precisely and their generated behavior can be examined and validated qualitatively and quantitatively with respect to experimental data.

Most models of PI favor a particular coordinate system (Cartesian or polar) and reference frame (geo- or egocentric) to perform PI based on theoretical and biological arguments (Vickerstaff and Cheung, 2010). While some models (Müller and Wehner, 1988; Hartmann and Wehner, 1995) include behavioral data from navigating animals in order to argue for their proposed PI method, others (Wittmann and Schwegler, 1995; Haferlach et al., 2007; Kim and Lee, 2011) have applied neural network models to investigate possible memory mechanisms for PI. Despite the wide variety of models, only a few of these models have been implemented on embodied artificial agents (Schmolke et al., 2002; Haferlach et al., 2007) and in foraging tasks similar to the ones faced by animals in terms of distance and tortuosity of paths (Lambrinos et al., 1997, 2000). Furthermore, while some vertebrate-inspired models (Gaussier et al., 2000; Jauffret et al., 2015) offer underlying spatial learning mechanisms based on place and view cells, many insect-inspired models have not linked PI and navigational capabilities to spatial learning and memory. A notable exception is a recent model based on the *Drosophila* brain show impressive results to generate adaptive behaviors in an autonomous agent, including exploration, visual landmark learning, and homing (Arena et al., 2014). However, the model has not been explicitly shown to be scalable for long-distance central-place foraging as observed in social insects.

Kubie and Fenton (2009) proposed a PI model based on the summation of path segments with HD accumulator cells, which are individually tuned to different HDs and hypothesized to encode how far the animal traveled in this direction. These summated path vectors are then stored in a fixed memory structure called shortcut matrix, which is used for navigating toward goals. Although this model is based on HD cells and therefore presented as for mammalian navigation, recent findings in *Drosophila melanogaster* (Seelig and Jayaraman, 2015) demonstrate that similar HD accumulator cells can also be hypothesized for insect navigation. Similar HD accumulator models have been applied for chemo-visual robotic navigation (Mathews et al., 2009) and PI-based homing behavior (Kim and Lee, 2011).

Cruse and Wehner (2011) presented a decentralized memory model of insect vector navigation to demonstrate that the observed navigational capabilities do not require a map-like memory representation. Their model is a cybernetical network structure, which mainly consists of a PI system, multiple memory banks and internal motivational states that control the steering angle of a simulated point agent. The PI system provides the position of the agent given by euclidean coordinates, which are stored as discrete vector memories when the agent finds a food location. To our knowledge, this model is the first and only modeling approach which accounts for behavioral aspects of insect vector navigation. However, although they introduce a learning rule for so-called quality values of stored vectors in a more recent version of the model (Hoinville et al., 2012), their model does not account for how the navigation vectors are represented and learned in a neural implementation.

### 1.4. Our approach

Inspired by these findings, in this paper, we present a novel model framework for PI and adaptive vector navigation as observed in social insects. The framework is applied as closed-loop control to an artificial agent and consists of four functional subparts: (1) a neural PI mechanism, (2) a reward-modulated learning rule for vector memories, (3) random search, and (4) an adaptive action selection mechanism. Here, the artificial agent primarily enables us to provide the necessary physical embodiment (Webb, 1995) in order to test the efficacy of our adaptive navigation mechanism, without a detailed reverse engineering of the insect brain.

Based on population-coded heading directions in circular arrays, we apply PI by accumulating speed-modulated HD signals through a self-recurrent loop. The final home vector representation is computed by local excitation-lateral inhibition connections, which projects accumulated heading directions onto the array of output neurons. The activity of these neurons encodes the vector angle as the position of maximum firing in the array, and the vector length as the amplitude of the maximum firing rate in the array. The self-localization ability of PI allows social insects to learn spatial representations for navigation (Collett et al., 1999). We design a reward-modulated associative learning rule (Smith et al., 2008; Cassenaer and Laurent, 2012; Hige et al., 2015) to learn vector representations based on PI. This vector, called global vector, connects the nest to a rewarding food location. Vectors are learned by associating the PI state and a reward received at the food location given a context-dependent state. This association induces weight changes in plastic synapses connecting the context-dependent unit to a circular array of neurons, which represents the vector. The context-dependent unit activates the vector representation in the array, and therefore represents a motivational state for goal-directed foraging. Using the vector learning rule, the agent is able to learn rewarding locations and demonstrate goal-directed navigation. Because of the vector addition of global and inverted home vector in the action selection mechanism, it can compensate for unexpected detours from the original trajectory, such as obstacles (Collett et al., 1999, 2001).

Taken together, our model is a novel framework for generating and examining social insect navigation based on PI and vector representations. It is based on plausible neural mechanisms, which are related to neurobiological findings in the insect central complex. Therefore, we provide a computational approach for linking behavioral observations to their possible underlying neural substrates. In the next section, we will describe the proposed model for reward-modulated vector learning and navigation. The results section will provide detailed descriptions of our experimental setups and simulation results. Finally, conclusions and implications of our model with respect to behavioral and neurobiological studies are discussed in Section 4.

## 2. Materials and methods

**Figure 1:**
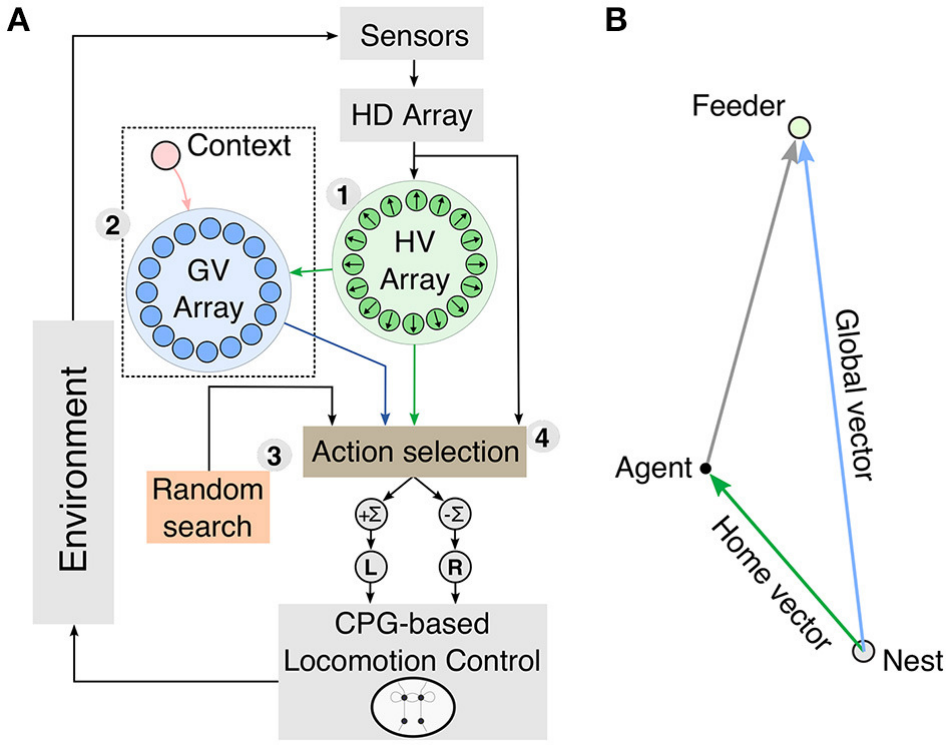
**Schematic diagram of the modular closed-loop control for vector navigation**. **(A)** The model consists of a neural path integration (PI) mechanism (1), reward-modulated vector learning (2), random search (3), and action selection (4). Vector information for guiding navigation is computed and represented in the activity of circular arrays. The home vector (HV) array is the output of the PI mechanism and is applied for homing behavior and as a scaffold for global vector (GV) learning. These three vector representations and random search are integrated through an adaptive action selection mechanism, which produces the steering command to the CPG-based locomotion control. **(B)** Spatial representation of the different vectors used for navigation. The HV is computed by PI and gives an estimate for the current location of the agent. In general, GVs connect the nest to a rewarding location. Using vector addition, the agent is able to compute, how to orient from its current location toward the feeder.

In this paper we propose an insect-inspired model of vector-guided navigation in artificial agents using modular closed-loop control. The model (see Figure 1A) consists of four parts: (1) a neural PI mechanism, (2) plastic neural circuits for reward-based learning of vector memories, (3) random search, and (4) action selection. The neural mechanisms in our model receive multimodal sensory inputs from exteroceptive and proprioceptive sensors to produce a directional signal based on a vector (see Figure 1B). This vector is represented by the activity of circular arrays, where the position of the maximum indicates its direction and the amplitude at this position indicates its length. We evaluate our model in simulation using a two-dimensional point agent as well as a hexapod walking robot (see Supplementary Material for details).

### 2.1. Path integration (PI) mechanism for home vector (HV) representation

**Figure 2:**
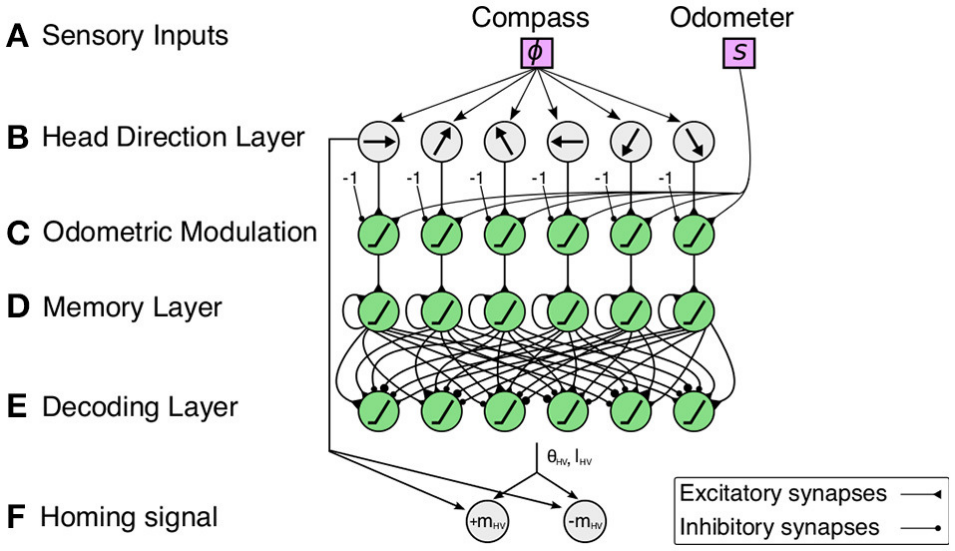
**Multilayered neural network of the proposed path integration (PI) mechanism**. **(A)** Sensory inputs from a compass sensor (ϕ) and odometer (*s*) are provided to the mechanism. **(B)** Neurons in the head direction (HD) layer encodes the sensory input from a compass sensor using a cosine response function. Each neuron encodes a particular preferred direction enclosing the full range of 2π. Note that the figure depicts only six neurons for simplicity. **(C)** An odometric sensory signal (i.e., walking speed) is used to modulate the HD signals. **(D)** The memory layer accumulates the signals by self-recurrent connections. **(E)** Cosine weight kernels decode the accumulated directions to compute the output activity representing the home vector (HV). **(F)** The difference between the HV angle and current heading angle is used to compute the homing signal (see Equation 11).

The PI mechanism (Figure 2) is a multilayered neural network consisting of circular arrays, where the final layer's activity pattern represents the HV. Neural activities of the circular arrays represent population-coded compass information and rate-coded linear displacements. Incoming signals are sustained through leaky neural integrator circuits, and they compute the HV by local excitatory-lateral inhibitory interactions.

**A) Sensory inputs**

The PI mechanism receives angular and linear cues as sensory inputs. Like in social insects, angular cues are derived from allothetic compass cues. We employ a compass sensor which measures the angle ϕ of the agent's orientation. In insects, this information is derived from the combination of sun- and skylight compass information (Wehner, 2003). In desert ants, it has been found that linear cues are derived from the strides taken by the animal during the journey (Wittlinger et al., 2006, 2007). For our model, we assume that such odometry is translated into an estimate of the animal's walking speed. For the embodied agent employed here (i.e., a hexapod robot), the walking speed is computed by accumulating steps and averaging over a certain time window. These step counting signals are derived from the motor signals. The input signals for the angular component ϕ and the linear component *s* have value ranges of

(1) $$\begin{array}{l} \phi \in \left[0,2\pi \right), \end{array}$$

(2) $$\begin{array}{l} s\in \left[0,1\right]. \end{array}$$

**B) Head direction layer**

The first layer of the neural network is composed of HD cells with activation functions

(3) $$\begin{array}{l} x_{i}^{\text{HD}}\left(\phi \left(t\right)\right)=\cos\left(\phi \left(t\right)-\phi_{i}\right), \end{array}$$

(4) $$\begin{array}{l} \phi_{i}=\frac{2\pi i}{N},i\in \left[0,N-1\right], \end{array}$$

where the compass signal ϕ(*t*) is encoded by a cosine response function with *N* preferred directions ϕ_*i*_ ∈ [0, 2π). The resolution is determined by $\Delta \phi =\frac{2\pi }{N}$ and the coarse encoding of variables, here angles, by cosine responses allows for high accuracy and optimized information transfer (Eurich and Schwegler, 1997). Coarse coding has been shown to be present in different sensory processing in the insect brain, including olfactory (Friedrich and Stopfer, 2001) and visual processing (Wystrach et al., 2014). Furthermore, it has been shown that polarization-sensitive neurons in the anterior optic tubercle of locusts exhibit broad and sinusoidal tuning curves of 90–120° (Heinze et al., 2009; Heinze and Homberg, 2009; el Jundi and Homberg, 2012). Head-direction cells in the central complex of *Drosophila melanogaster* were shown to have activity bump widths of 80–90° (Seelig and Jayaraman, 2015). However, their measurements are based on calcium imaging data, which is only an approximation of the neuron's firing rate.

**C) Odometric modulation of head direction signals**

The second layer acts as a gating mechanism (G), which modulates the neural activity using the odometry signal *s* (∈[0, 1]). Therefore, it encodes in its activity, the traveled distances of the agent. The gating layer units decrease the HD activities by a constant bias of 1, so that the maximum activity is equal to zero. A positive speed increases the signal linearly. The gating activity is defined as follows:

(5) $$\begin{array}{l} x_{i}^{\text{G}}\left(t\right)=f\left(\overset{N-1}{\underset{j\;=\;0}{\sum }}\delta_{ij}x_{j}^{HD}\left(t\right)-1+s\right), \end{array}$$

(6) $$\begin{array}{l} f\left(x\right)=\max\left(0,x\right), \end{array}$$

where δ_*ij*_ is the Kronecker delta, i.e., first layer neurons *j* and second layer neurons *i* are connected one-to-one. Forward speed signals have been found in the central complex of walking cockroaches (Martin et al., 2015).

**D) Memory layer**

The third layer is the so-called memory layer (M), where the speed-modulated HD activations are temporally accumulated through self-excitatory connections:

(7) $$\begin{array}{l} x_{i}^{\text{M}}\left(t\right)=f\left(\overset{N-1}{\underset{j\;=\;0}{\sum }}\delta_{ij}x_{j}^{G}\left(t\right)+\left(1-\lambda \right)x_{i}^{\text{M}}\left(t-\Delta t\right)\right), \end{array}$$

where λ is a positive constant defined as the integrator leak rate, which indicates the loss of information over time. A leaky integrator has previously been applied by Vickerstaff (2007) to explain systematic errors in homing of desert ants (Müller and Wehner, 1988). If the leak rate is equal to zero, the accumulation of incoming directional signals is unbounded, which is not biologically plausible. As such, any path integration system based on linear integration therefore bounds the natural foraging range of the animal in order to exhibit accurate path integration (Burak and Fiete, 2009).

**E) Decoding layer**

**Figure 3:**
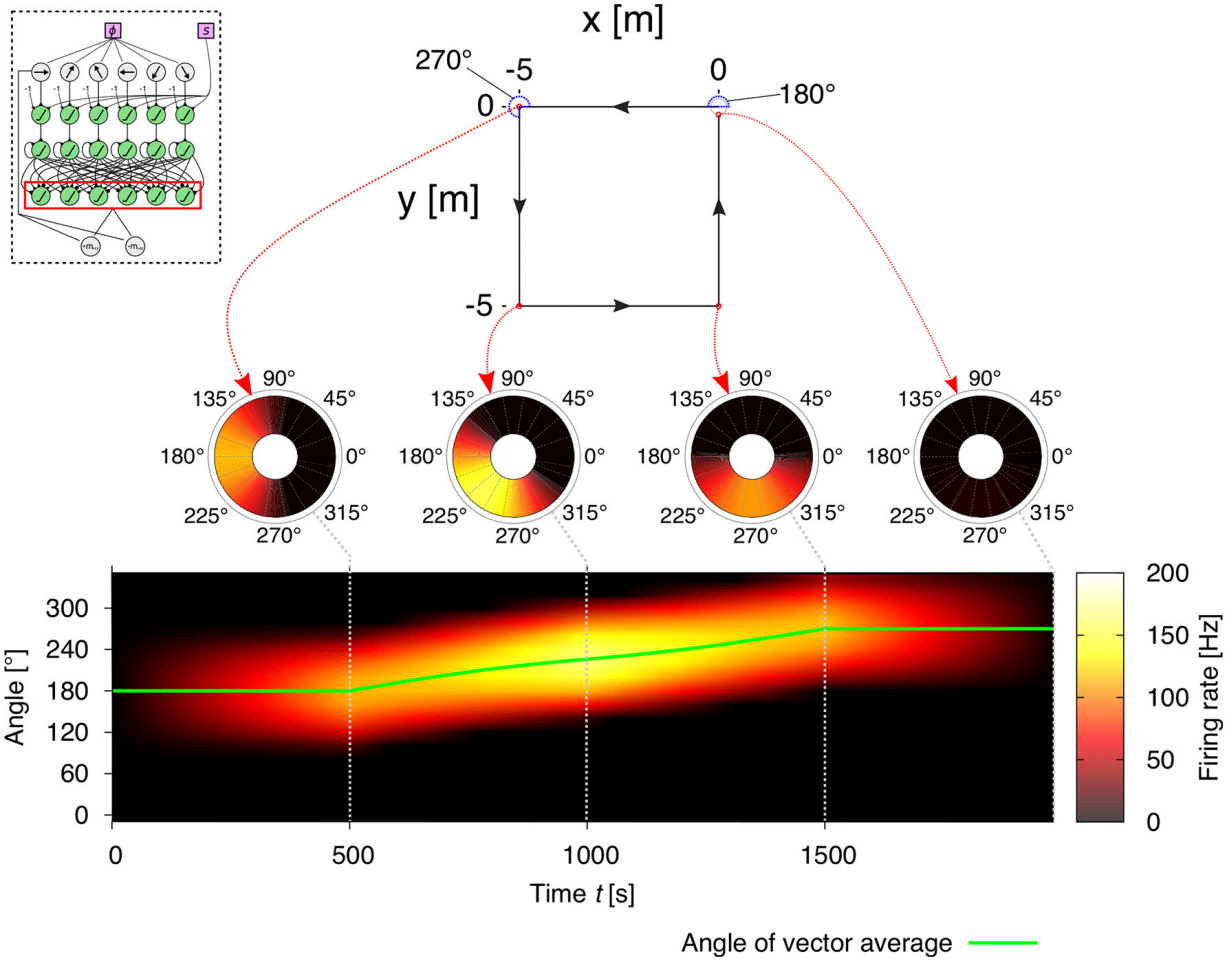
**Example of vector representations based on the neural activities of the decoding layer (see Figure **2E**) in the path integration (PI) mechanism for a square trajectory**. The agent runs for 5 m in one of the four directions (180°, 270°, 0°, 90°), thus finally returning to the starting point of its journey. The coarse encoding of heading orientations lead to a correct decoding of memory layer activities. Thus, the activities of the decoding layer in the PI mechanism (see inlay) represent the home vector (HV), where the position of the maximum firing rate is the angle and the amplitude of the maximum firing rate is the length of the vector. Note that, as the agent returns to the home position, the output activities are suppressed to zero resulting from the elimination of opposite directions.

The final and fourth layer decodes the activations from the memory layer to produce a vector representation, i.e., the HV, which serves as the output of the mechanism referred to as PI state:

(8) $$\begin{array}{l} x_{i}^{\text{PI}}\left(t\right)=f\left(\overset{N-1}{\underset{j\;=\;0}{\sum }}w_{ij}x_{j}^{\text{M}}\left(t\right)\right) \end{array}$$

(9) $$\begin{array}{l} w_{ij}=\cos\left(\phi_{i}-\phi_{j}\right)=\cos\left(\frac{2\pi \left(i-j\right)}{N}\right), \end{array}$$

where *w*_*ij*_ is a cosine kernel, which decomposes the projections of memory layer actitivities of the *j*th neuron to the *i*th neuron's preferred orientation. While a cosine synaptic weight kernel is biologically implausible, it is reasonable to assume that an approximate connectivity could arise from forming local-excitation lateral-inhibition connections (e.g., mexican-hat connectivity). An example of such a connectivity formed by cell proximity could be the ring architecture of head-direction-selective neurons in the ellipsoid body of the central complex (Seelig and Jayaraman, 2015; Wolff et al., 2015). The resulting HV is encoded by the average position of maximum firing in the array (angle θ_*HV*_) and the sum of all firing rates of the array (length *l*_*HV*_). We calculate the position of maximum firing using the population vector average given by:

(10) $$\begin{array}{l} \theta_{HV}\left(t\right)=\arctan\left(\frac{\sum_{i\;=\;0}^{N-1}x_{i}^{\text{PI}}\left(t\right)\;\sin\;\left(2\pi i/N\right)}{\sum_{i\;=\;0}^{N-1}x_{i}^{\text{PI}}\left(t\right)\cos\left(2\pi i/N\right)}\right), \end{array}$$

where the denominator is the *x* coordinate of the population vector average, and the numerator is the *y* coordinate. See Figure 3 for example output activities of the decoding layer neurons.

**F) Homing signal**

To apply the HV for homing behavior, i.e., returning home on a straight path, the vector is inverted by a 180° rotation. The difference between the heading direction ϕ and the inverted HV direction θ_*HV*_−π is used for steering the agent toward home. The agent applies homing by sine error compensation, which defines the motor command:

(11) $$\begin{array}{l} m_{HV}(t)=l_{HV}(t)\;\sin\;\left(\theta_{HV}(t)-\phi (t)-\pi \right) \end{array}$$

This leads to right (*m*_*HV*_ < 0) and left turns (*m*_*HV*_ > 0) for negative and positive differences, respectively, and thereby decreasing the net error at each step. The underlying dynamical behavior of this sine error compensation is defined by a stable and an unstable fixed point (see Supplementary Marterial). This leads to dense searching behavior around a desired position, where the error changes rapidly (Vickerstaff and Cheung, 2010).

### 2.2. A reward-modulated learning rule for acquiring and retrieving vector memories

**Figure 4:**
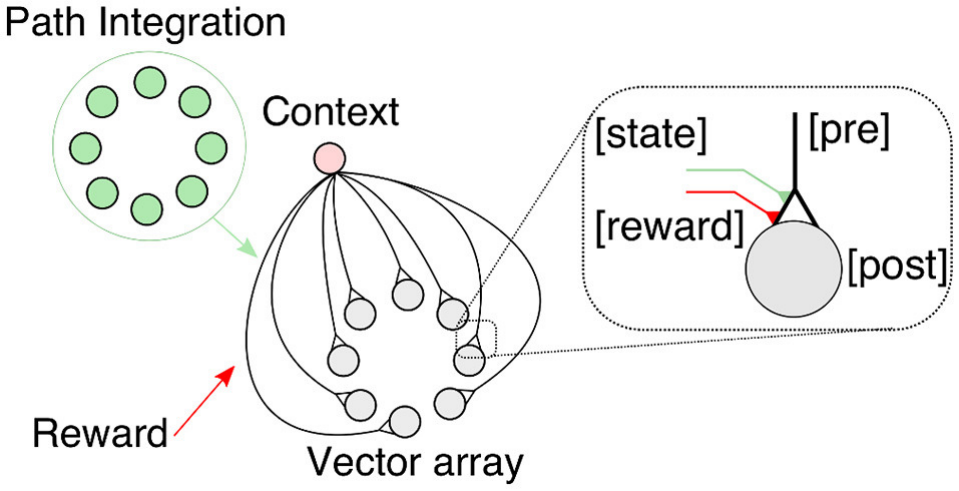
**Canonical vector learning rule involves associations of path integration (PI) states with context-dependent and reward signals**. Global vector memories are acquired and expressed by this learning circuit. The home vector array activities are associated with the food reward given an active foraging state (outward journey). For details, see text below.

We propose a heterosynaptic, reward-modulated learning rule (Smith et al., 2008; Cassenaer and Laurent, 2012; Hige et al., 2015) with a canonical form to learn vector memories based on four factors (see Figure 4): a context-dependent state, an input-dependent PI state, a modulatory reward signal, and the vector array state. Like the HV, GV memories are computed and represented in circular arrays. The context-dependent state, such as inbound or outbound foraging, activates the vector representation, and thus retrieves the vector memory. The association between the PI-based state and the reward signal modulates the plastic synapses connecting the context unit (presynaptic) with the vector array units (postsynaptic). The associated information is used by the agent on future foraging trips to steer toward the rewarding location. The received reward is an internally generated signal based on food reward due to visiting the feeder.

The context-dependent unit (see Figure 4) is a unit that represents the agent's foraging state, i.e., inward or outward. Here we apply a simple binary unit given by:

(12) $$\begin{array}{l} \sigma \left(t\right)=\left\{ \begin{array}{cc} 1\; & \text{if outward trip},\\ 0\; & \text{if inward trip}. \end{array}\right. \end{array}$$

The context-dependent unit projects plastic synapses onto a circular array that represents the GV. The GV array has the same number of neurons, thus the same preferred orientations as the PI array. In this way, each neuron *i* ∈ [0, *N* − 1] has a preferred orientation of $\frac{2\pi i}{N}$. The activity $x_{i}^{GV}$ of the GV array is given by:

(13) $$\begin{array}{l} x_{i}^{GV}\left(t\right)=w_{i}^{GV}\left(t\right)\sigma \left(t\right), \end{array}$$

where $w_{i}^{GV}$ are the weights of the plastic synapses. For these synapses, we apply a reward-modulated associative learning rule given by:

(14) $$\begin{array}{l} \Delta w_{i}^{GV}\left(t\right)=\mu^{GV}r\left(t\right)\sigma \left(t\right)\left(x_{i}^{PI}\left(t\right)-x_{i}^{GV}\left(t\right)\right), \end{array}$$

(15) $$\begin{array}{l} w_{i}^{GV}\left(t+\Delta t\right)=w_{i}^{GV}\left(t\right)+\Delta w_{i}^{GV}\left(t\right), \end{array}$$

where μ^*GV*^ = 2 is the learning rate, and $x_{i}^{PI}\left(t\right)$ is the PI activity in the direction $i=\frac{2\pi i}{N}$. The weights are therefore only changed when the agent forages outbound, because for the inward trip we assume that the agent returns to the home on a straight path. This is in accordance with behavioral data indicating that ants acquire and retrieve spatial memories based on internal motivational states, given by whether they are on an inward or outward trip (Wehner et al., 2006). The food reward *r*(*t*) at the feeder is given by:

(16) $$\begin{array}{l} r\left(t\right)=\max\left(0,1-5d\left(t\right)\right) \end{array}$$

where *d*(*t*) is the agent's distance to the feeder, which we computed directly using the positions of the agent and feeder, given that the reward is physically bound to the location of the food. Due to the delta rule-like term $x_{i}^{PI}\left(t\right)-x_{i}^{GV}\left(t\right)$, the weights $w_{i}^{GV}$ approach same values as the activities of the PI state at the rewarding location. Thus, the weights represent the static GV to the rewarding location (feeder). After returning back home, the agent applies the angle θ_*GV*_ of the GV to navigate toward the feeder using error compensation. The motor signal of the GV:

(17) $$\begin{array}{l} m_{GV}(t)=l_{GV}(t)\;\sin\;\left(\theta_{GV}(t)-\phi (t)\right), \end{array}$$

is applied together with the homing signal *m*_*HV*_ and random search *m*_ε_, where *l*_*GV*_ is the length of the GV. We model the random search by the agent as a correlated Gaussian random walk, which has been previously used to study animal foraging (Bovet and Benhamou, 1988). Therefore, *m*_ε_ is drawn from a Gaussian distribution N (mean, S.D.):

(18) $$\begin{array}{l} m_{\varepsilon }\left(t\right)\in N\left(0,\varepsilon \left(t\right)\right), \end{array}$$

with an adaptive exploration rate ε(*t*) given by:

(19) $$\begin{array}{l} \varepsilon (t)=\sigma (t)\;\exp\;\left(-\beta (t)v(t)\right), \end{array}$$

where *v*(*t*) is an estimate for the average food reward received over time and β(*t*) is the inverse temperature parameter. The exploration rate is thus zero for inward trips, because the agent applies path integration to reach its home position on a straight path. We define *v* by the recursive formula:

(20) $$\begin{array}{l} v\left(t\right)=r\left(t\right)+\gamma v\left(t-\Delta t\right), \end{array}$$

where *v*(*t*) is a lowpass filtered signal of the received food reward *r*(*t*) with discount factor γ = 0.995. Convergence of goal-directed behavior is achieved for ε below a critical value, which depends on the choice of β. We assume that ϵ and *v* are based on a probability distribution with fixed mean. We derive a gradient rule, which leads to minimization of the Kullback-Leibler divergence between the distribution of ϵ(*v*) and an optimal exponential distribution (see Supplementary Material for a derivation). The learning rule is given by:

(21) $$\begin{array}{l} \Delta \beta \left(t\right)=\mu_{\beta }\left(\frac{1}{\beta \left(t\right)}+\mu_{v}v\left(t\right)\varepsilon \left(t\right)\right), \end{array}$$

(22) $$\begin{array}{l} \beta \left(t+\Delta t\right)=\beta \left(t\right)+\Delta \beta \left(t\right), \end{array}$$

where $\mu_{\beta }=10^{-6}$ is a global learning rate, $\mu_{v}=10^{2}$ is a reward-based learning rate. The adaptation of beta is characterized by small changes scaling with the square root of time, while the term containing *v*(*t*) allows for exploitation of explored food rewards to further decrease ε through β. In ecological terms, such exploitation of sparse distributed resources is crucial for the survival of an individual as well as the whole colony (Biesmeijer and de Vries, 2001; Wolf et al., 2012; Bolek and Wolf, 2015).

The final motor command Σ in our action selection mechanism is given by the linear combination:

(23) $$\begin{array}{l} \Sigma (t)=\left(1-\varepsilon (t)\right)\left(\sigma (t)m_{GV}(t)+m_{HV}(t)\right)+m_{\varepsilon }(t), \end{array}$$

where outward trips are controlled by the balance of random walk and global-vector guided navigation depending on the exploration rate ε, while inward trips are controlled solely by the homing signal *m*_*HV*_. The combination of the two sinusoidals is equivalent to a phase vector (phasor) addition resulting in a phasor, which connects the current position of the agent with the learned feeder location (see Supplementary Material for a derivation).

## 3. Results

Using the proposed model embedded as a closed-loop control into a simulated agent, we carried out several experiments to validate the performance and efficiency in navigating the agent through complex and noisy environments. We will further demonstrate that the generated behaviors not only resemble insect navigational strategies, but can also predict certain observed behavioral parameters of social insects.

### 3.1. Path integration (PI) in noisy environments

It has been shown, both theoretically and numerically, that PI is inherently prone to error accumulation (Benhamou et al., 1990; Vickerstaff and Cheung, 2010). Studies have focused on analyzing resulting errors from using certain coordinate systems to perform PI (Benhamou et al., 1990; Cheung and Vickerstaff, 2010; Cheung, 2014). Here we apply a system of geocentric static vectors (fixed preferred orientations) and analyze the effect of noise on the resulting error. How can noise be characterized in PI systems? Both artificial and biological systems operate under noisy conditions. Artificial systems, such as robots employ a multitude of sensors which provide noisy measurements, and generate motor outputs that are similarly noisy. Rounding errors in their control systems can be an additional source of noise. In animals, noise is mainly attributed to random influences on signal processing and transmission in the nervous system, including synaptic release and membrane conductance by ion channels and pumps (see Stein et al., 2005 for review).

**Figure 5:**
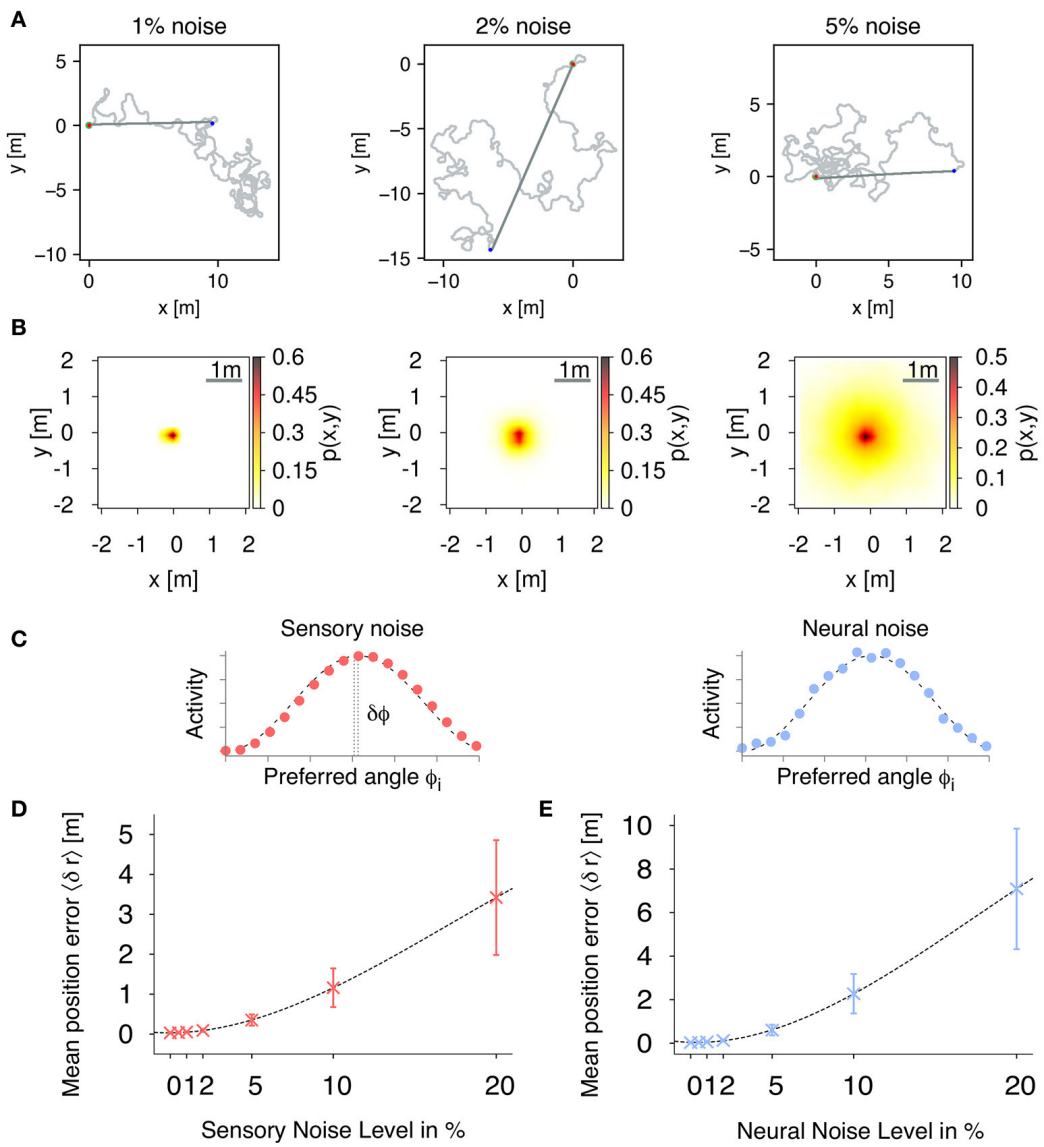
**Path integration (PI) accuracy under the influence of external noise**. **(A)** Example trajectories of the simulated agent during random foraging (light gray) and homing behavior (dark gray) for different sensory, correlated noise levels: 1, 2, and 5%. The red point marks the starting point at the nest, and the blue point indicates the return, when the agent switches to its inward state. Using only path integration, the agent successfully navigates back to the nest with a home radius (green circle) of 0.2 m. **(B)** We evaluate the accuracy of the proposed PI mechanism by using the mean positional error averaged over each time step during each trial. Distribution of positional errors for different sensory, correlated noise levels: 1, 2, and 5%. **(C)** Examples of population-coded HD activities with correlated and uncorrelated noise. Filled dots are activities of individual neurons, while the dashed line is a cosine response function. **(D)** Mean position errors 〈δ*r*〉 (± S.D.) in PI with respect to fully correlated, sensory noise levels averaged over 1,000 trials (fixed number of 18 neurons per layer). **(E)** Mean position errors 〈δ*r*〉 (± S.D.) in PI with respect to uncorrelated, neural noise levels averaged over 1,000 trials (fixed number of 18 neurons per layer).

In order to validate the accuracy of the PI mechanism, we measure the positional errors of the estimated nest position with respect to the actual position over time. In the following experiments, we averaged positional errors over 1,000 trials with trial duration *T* = 1, 000 s (simulation time step Δ*t* = 0.1 s). In each trial, the agent randomly forages out from the nest and when the trial duration *T* is reached, the agent switches to the inward state and only applies the path integration mechanism for homing (see Figure 5A for example trajectories). After trial duration *T*, the mean distance of the agent from the nest is 9.3 ± 5.0 m. The radius of the nest the agent has to reach for successful homing is set to 20 cm. Figure 5B shows the distribution of positional errors for three different correlated, sensory noise levels (1, 2, and 5%). The distribution of errors follows a two-dimensional Gaussian distribution with mean 0.0 (nest) and width 〈δ*r*〉.

In population coding, neural responses are characterized by correlated or uncorrelated noise (Averbeck et al., 2006, see Figure 5C for examples). In the uncorrelated case, fluctuations in one neuron are independent from fluctuations in the other neurons. Correlated noise is described by fluctuations which are similarly expressed across the population activity, and therefore leads to a shift of the observed peak activity. Here, we numerically analyze the effects of correlated and uncorrelated noise on the accuracy of the proposed PI mechanism. Correlated noise is here defined as a shift δϕ of the peak activity, i.e., fully correlated noise, such that the compass input to the PI mechanism is given by:

(24) $$\begin{array}{l} \phi_{noisy}\left(t\right)=\phi \left(t\right)+\delta \phi , \end{array}$$

where δϕ is drawn from a Gaussian distribution $N\left(0,2\pi \zeta_{sens}\right)$ with sensory noise level ζ_*sens*_. Uncorrelated noise, also referred to as neural noise, is defined by adding fluctuations $\delta x_{i}^{HD}$ to the activities of the HD layer, which are drawn from a Gaussian distribution $N\left(0,\zeta_{neur}\right)$ with neural noise level ζ_*neur*_.

Figure 5D shows the effect of different degrees of sensory noise on the performance of PI for a fixed number of 18 neurons per layer averaged over 1000 trials. For noise levels up to 5% (equal to 18°), the observed mean position error increases only slowly and nonlinearly with values below 0.4 m demonstrating that our PI mechanism is robust for sensory noise up to these levels.

In Figure 5E, we show mean position errors for different levels of uncorrelated noise. Similar to sensory noise, the errors first increase slowly and nonlinearly for noise up to 2%, while for noise larger than 5%, errors increase linearly. In comparison with sensory noise levels, uncorrelated noise leads to larger errors due to a more dispersed peak activity. However, for noise levels up to 2%, mean position errors are well below 0.2 m indicating robustness of our PI mechanism with respect to uncorrelated noise. Given this apparent similar nature of correlated and uncorrelated noise, we only applied sensory, correlated noise for the following experiments of this study.

**Figure 6:**
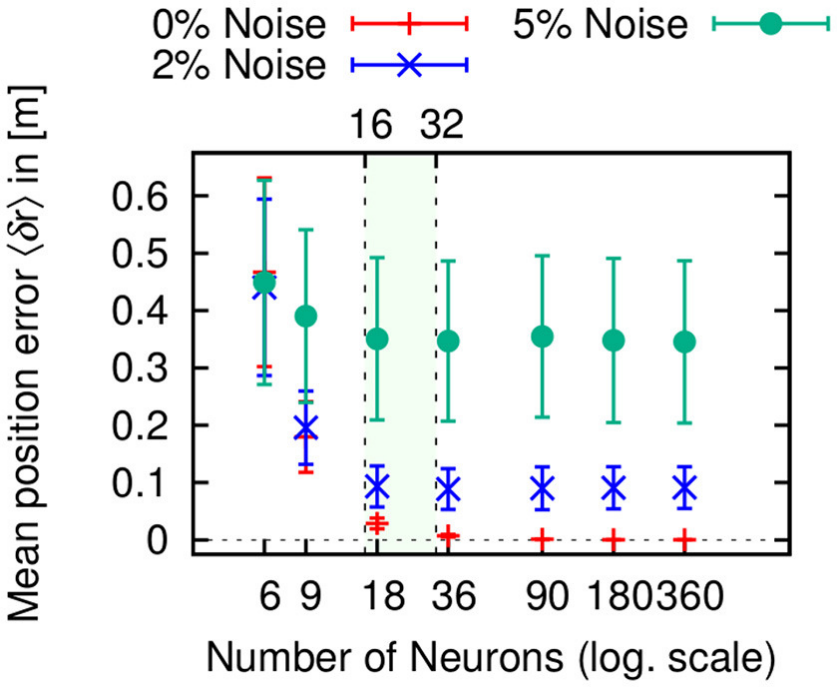
**Mean positional errors 〈δ***r***〉 (± S.D.) in path integration (PI) with respect to number of neurons per layer averaged over 1, 000 trials for three different sensory noise level (0, 2, and 5%)**. In all three cases, the error reaches a minimum plateau between 16 and 32 neurons (colored area), which corresponds to the number of functional columns in the ellipsoid body of the insect central complex (Wolff et al., 2015).

In Figure 6, we varied the number of neurons in the circular arrays of the PI mechanism for three different sensory noise level (0, 2, and 5%). Note that the errors for 0% noise arise from the accuracy limit given the number of neurons. While the mean position error is significantly higher for 6 and 9 neurons, it achieves a minimal value for 18 neurons. For larger system sizes, the error only changes minimally. This is again mainly due to the coarse coding of heading directions. Interestingly, the ellipsoid body of the insect central complex contains neurons with 16–32 functional arborization columns (called wedges, see Wolff et al., 2015). The numerical results here might point toward an explanation for this number, which efficiently minimizes the error.

**Figure 7:**
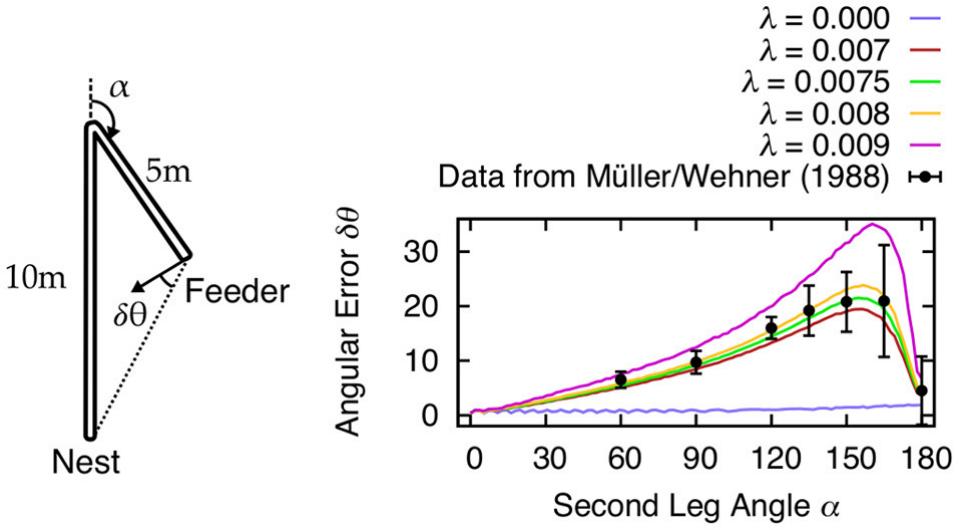
**Systematic errors δθ of desert ant homing are reproduced by leaky integration of path segments**. Müller and Wehner (1988) tested the ants how accurate they return to the nest after following the two connected, straight channels with 10 and 5 m length to the feeder (sketch modified from Müller and Wehner, 1988). The second channel angle α was varied in 2.5° intervals for the simulation results. In our model, the leak rate λ in the self-recurrent connections is used to fit the behavioral data (Müller and Wehner, 1988). We found that values λ ≈ 0.0075 accurately describe the observed systematic errors in desert ants.

Besides errors resulting from random noise, there are also systematic errors observed in navigating animals. Both invertebrate and vertebrate species exhibit systematic errors in homing behavior after running an L-shaped outward journey (see Etienne and Jeffery, 2004 for review). Müller and Wehner (1988) have examined such errors in desert ants by measuring the angular deviation with respect to the angle of the L-shaped course (see Figure 7). In order to show that our mechanism is able to reproduce these errors, we fit our model against the desert ant data from Müller and Wehner (1988) using the leak rate λ (Equation 7) of the PI memory layer as control variable. Using a leak rate of λ ≈ 0.0075 resulted in angular errors most consistent with behavioral data. Leaky integration producing systematic errors is an idea that has been previously proposed (Mittelstaedt and Glasauer, 1991; Vickerstaff and Cheung, 2010). Thus, here our mechanism is not only performing accurately in the presence of random noise, but it also reproduces behavioral aspects observed in animals.

In Table 1, we compare the accuracy and efficiency with other state-of-the-art PI models. Haferlach et al. (2007) apply less neurons than our model, but we achieve a better performance in terms of positional accuracy with larger sensory noise (values taken from **Figure 9**). Note that our model achieves similar accuracy, when using six neurons (see Figure 6). The model by Kim and Lee (2011) applies 100 neurons per layer leading to a fairly small positional error despite of 10% uncorrelated noise (Figure 6A, *N*_1_ = 100 neurons). However, both models apply straight paths before homing, which results in smaller path integration errors compared to random foraging as observed in insects. Furthermore, many desert ant species were measured to freely forage average distances of 10–40 m depending on the species (Muser et al., 2005), whereas some individuals travel even up to multiple hundred meters (Buehlmann et al., 2014). Our foraging time has been adjusted for realistic foraging distances, and if we reduce the foraging time in our model, we achieve similarly small positional errors as previous models. Furthermore, behavioral data measured in desert ants (Merkle et al., 2006) revealed that path integration errors are approximately 1–2 m depending on foraging distance. The median values are taken from Figure 3B in Merkle et al. (2006) and reflect the error between the endpoint of an ant's inward run and the correct position of the nest. These larger errors compared to model accuracies are likely due to noise accumulation in sensing, neural processing and motor control, although it is difficult to determine an exact quantification. Nonetheless, ants are able to reliably navigate by falling back to other strategies, such as searching behavior or visual homing.

**Table 1:** **Comparison of existing path integration (PI) models in terms of accuracy and efficiency**.

**Model**	**Neurons**	**Noise [%]**	**Error [m]**	**Foraging dist. [m]**
Haferlach et al., 2007	6	3	0.46 ± 0.18	≤ 5
Kim and Lee, 2011	100	10	0.018 ± 0.002	≤ 5
Our model	18	5	0.351 ± 0.140	9.3 ± 5.0
	18	10	1.160 ± 0.484	9.3 ± 5.0
	18	5	0.070 ± 0.037	5 ± 3
*Cataglyphis fortis*	–	–	median=1.27 (*N* = 51)	5
(Merkle et al., 2006)	–	–	median=2.45 (*N* = 53)	10
	–	–	median=2.47 (*N* = 50)	20

### 3.2. Global vector (GV) learning and goal-directed navigation

In the previous section, we proposed a reward-modulated associative learning rule for GV learning. In order to test the performance of our insect-inspired model applying this learning rule, and to validate the use of learned vector representation in goal-directed navigation, we carried out several experiments under biologically realistic conditions. We apply the PI mechanism with *N* = 18 neurons per layer and a sensory noise level of 5%. In the first series of experiments, a single feeder is placed with a certain distance *L*_*feed*_ and angle θ_*feed*_ to the nest. The agent is initialized at the nest with a random orientation drawn from a uniform distribution on interval [0, 2π). In this naïve condition, the agent starts to randomly search in the environment. If the agent is unsuccessful in locating the feeder after a fixed time *t*_*forage*_, it turns inward and performs homing behavior using only the PI mechanism. If the agent however finds the feeder, the current PI state is associated with the received reward, and stored in the weights to the GV array. The agent returns back home after the accumulated reward surpasses a fixed threshold. Each trial lasts a fixed maximum time of $T=\frac{3}{2}t_{forage}$, before the agent is reset to the nest position. On subsequent foraging trips, the agent applies the learned vector representation and navigates along the GV, because the exploration rate is decreased due to the previous reward. If the agent finds the feeder repeatedly, the learned GV stabilizes and the exploration rate decreases further.

**Figure 8:**
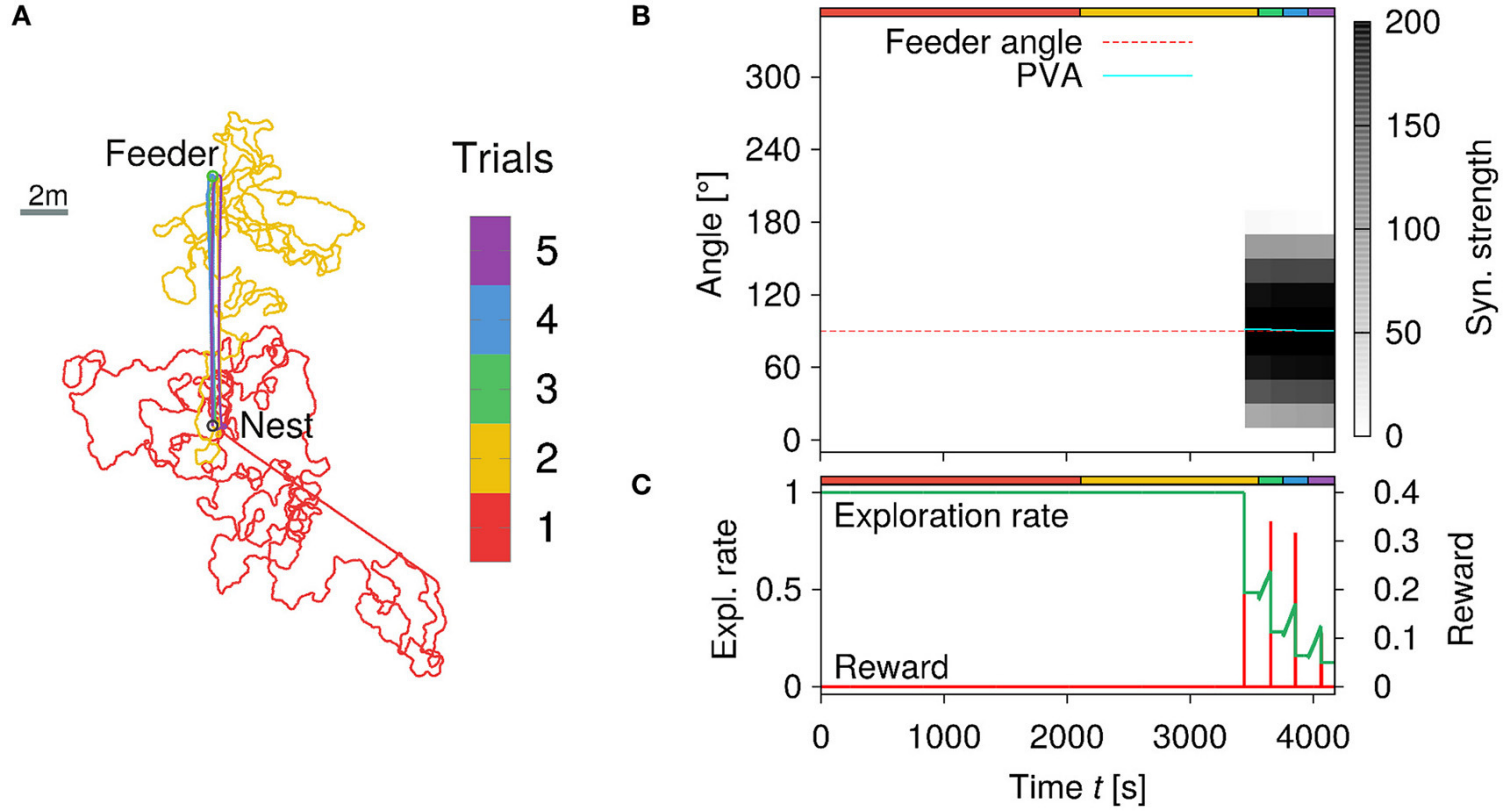
**Learning walks of the simulated agent for a feeder placed ***L***_***feed***_ = 10 m away from the nest. (A)** Trajectories of the agent for five trials with a feeder in 10 m distance and 90° angle to the nest. Each trial number is color-coded (see colorbar). Inward runs are characterized by straight paths controlled only by PI. See text for details. **(B)** Synaptic strengths of the GV array changes due to learning over time (of the five trials). The estimated angle θ_*GV*_ (cyan-colored solid line) to the feeder is given by the position of the maximum synaptic strength. **(C)** Exploration rate and food reward signal with respect to time. The exploration rate decreases as the agent repeatedly visits the feeder and receives reward.

Figure 8 demonstrates such an experiment for a feeder with a distance of *L*_*feed*_ = 10 m and angle $\theta_{feed}=90^{{}^{\circ}}$ from the nest. In Figure 8A, we show the trajectories of the agent during five trials. The trial numbers are color-coded (see colorbox). During the first trial, the agent has not visited the feeder yet and returns home after *t*_*forage*_ = 2, 000 s of random search. During the second trial (see yellow-colored trajectory), the agent finds the feeder and learns the GV representation from the PI state (see Figure 8B). Here the red dotted line indicates the correct angle $\theta_{feed}=90^{{}^{\circ}}$ to the feeder, while the cyan-colored line is the average angle estimated from the synaptic strengths of the GV array. In doing so, the agent is able to acquire an accurate vector representation (Figure 8B) resulting in stable trajectories toward the goal for the final three trials, which is again due to a low exploration rate (Figure 8C). The repeated visits to the feeder decrease the exploration rate due to the received reward (red line). In the final two trials, the agent navigates to the feeder on a stable trajectory (i.e., low exploration rate) demonstrating that the learning rule is robust for goal-directed navigation in noisy environments. Note, that the reward signal peak is decreased for the final two trials, because the agent does not enter the reward area centrally. Furthermore, switching the context unit to the inbound state is determined by the accumulated amount of reward over time. As such, smaller, but broader reward signals give a similar accumulated reward than a bigger and sharper signal.

**Figure 9:**
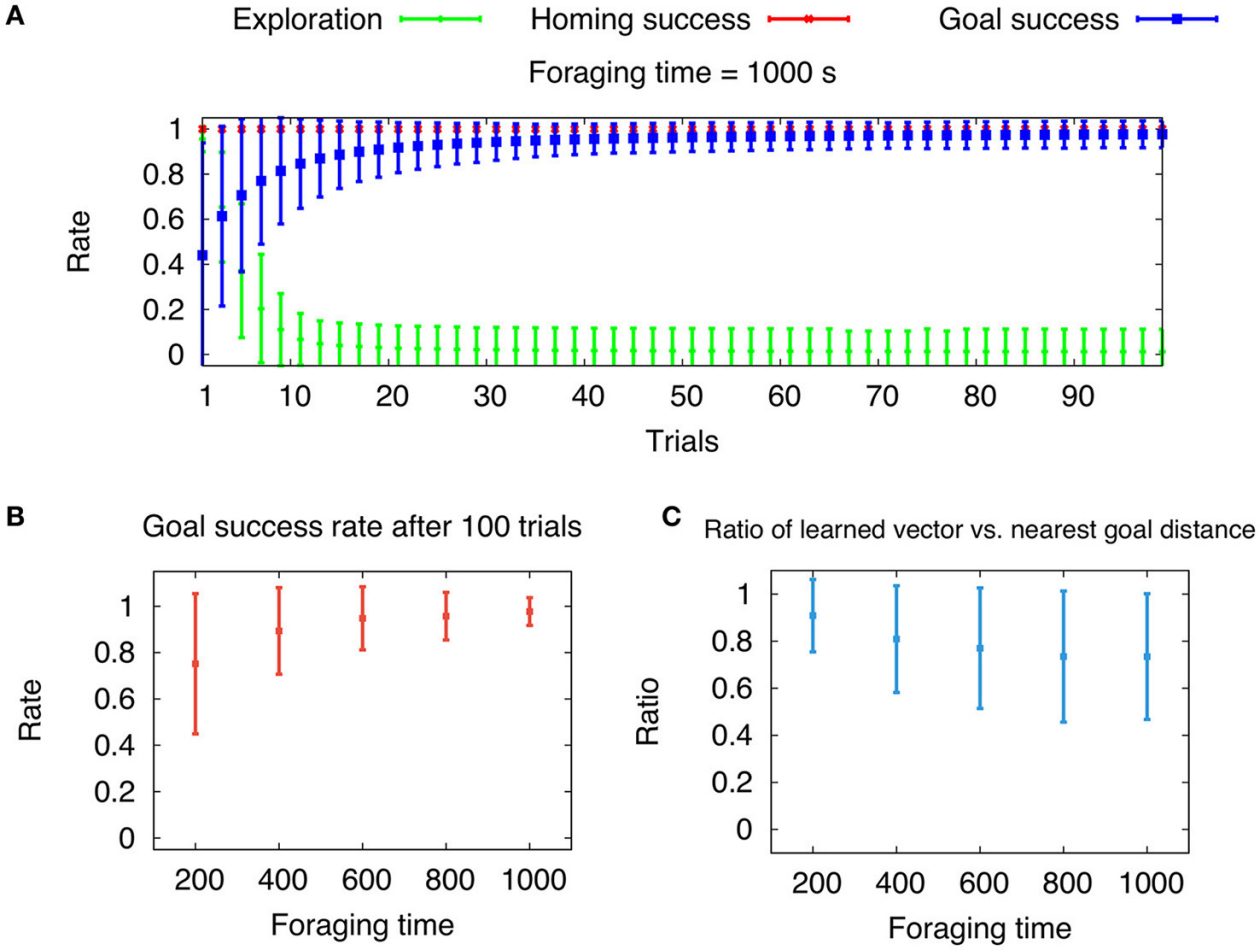
**Longer foraging durations during global vector (GV) learning increase the average goal success rate, but decrease the ratio of learned global vector and nearest feeder distance**. **(A)** Mean exploration rate and running mean goal success and homing rate (± S.D.) with respect to trials averaged over 100 cycles of randomly generated environments (foraging time *t*_*forage*_ = 1, 000 s). Goal success is defined by whether a feeder was visited per trial. The homing rate is determined by the agent's return to the nest within the given total trial duration *T*. **(B)** Mean goal success rate after 100 trials with respect to foraging time *t*_*forage*_ averaged over 100 cycles. **(C)** Mean ratio of learned GV distance and nearest feeder distance with respect to foraging time *t*_*forage*_ averaged over 100 cycles.

In Figure 9, we simulated 100 learning cycles with different randomly generated environments, each consisting of 100 consecutive trials. The feeders are randomly placed by sampling from a uniform distribution U as follows:

(25) $$\begin{array}{l} r_{feed}=\left(r_{max}-r_{min}\right)\sqrt{n_{1}}+r_{min}, \end{array}$$

(26) $$\begin{array}{l} \theta_{feed}=2\pi n_{2}, \end{array}$$

(27) $$\begin{array}{l} n_{1},n_{2}\in U\left(0,1\right), \end{array}$$

where *r*_*feed*_ is the distance from the nest to a feeder and θ_*feed*_ is the angle with respect to the x axis. We chose the *r*_*min*_ = 1 m and *r*_*max*_ = 40 m to be the bounds, in which the feeders can be placed. The density is determined by how many feeders will be placed within these bounds. Here, we generated 50 feeders for each environment. In Figure 9A, we show the mean exploration rate, and the running averages of mean homing and goal success rates with respect to trials (foraging time *t*_*forage*_ = 1, 000 s, averaged over 100 cycles). Note that the foraging time has been reduced compared to Figure 8, because the random environment contain multiple, not just a single feeder. This leads to a higher probability of finding a feeder and for the learning algorithm to converge. During the 100 trials, learning converges on average within the first 20 trials given by a low mean exploration rate. Like in the previous experiment, the agent reaches the feeder in every trial after convergence is achieved. This is indicated by the goal success approaching one. Average homing success is one for every trial, which results from sufficient searching behavior and the given total time *T*. The convergence of the learning process is dependent on the foraging time, because longer time allow for longer foraging distances, and thus larger search areas. Therefore, we varied the foraging time *t*_*forage*_ = 200, 400, 600, 800, and 1, 000 s and measure the mean goal success rate after 100 trials averaged over 100 cycles (Figure 9B). Note, that in contrast to naturalistic learning in ants, our agents reduces the exploration rate to zero leading to pure exploitation of the learned global vector. Ants live in environments with rather sparse, dynamic food sources, thus their exploitation of learned vector memories is rather flexible. Nevertheless, our results indicate that for longer foraging times, the mean goal success rate approaches one and its variance decreases. However, by measuring the averaged ratio of learned vector and nearest feeder distance, we show that this ratio decreases for larger foraging times (Figure 9C). Thus, there is a trade-off with respect to convergence and reward maximization, leading to an optimal foraging time. Desert ants have been shown to increase their foraging times up to a certain value, after which it saturates (Wehner et al., 2004). This adaptation of foraging time might be indicated by the trade-off resulting from our model. Furthermore, we encourage the reader to see the Supplementary Video of path integration and global vector learning performed by a simulated hexapod robot.

## 4. Discussion

Social insects, such as bees and ants, use PI-based vector memories for guiding navigation in complex environments (Collett et al., 1998, 1999; De Marco and Menzel, 2005; Collett and Collett, 2015). Here, we proposed a novel computational model for combining PI and the acquisition of vector memories in a simulated agent. We have shown that a computational model based on population-coded vector representations can generate efficient and insect-like navigational behaviors in artificial agents. These representations are computed and stored using a simple neural network model combined with reward-modulated associative learning rules. Thus, the proposed model is not only accounting for a number of behavioral aspects of insect navigation, but it further provides insights in possible neural mechanisms in relevant insect brain areas, such as the central complex. In the following, we will discuss certain aspects of our model juxtaposing it with neurobiological findings in insects. Furthermore, we provide comparisons to other state-of-the-art models of vector-guided navigation (Kubie and Fenton, 2009; Cruse and Wehner, 2011).

### 4.1. Head-direction (HD) cells and path integration (PI)

A main property of the PI mechanism of our model is that it receives input from a population of neurons, which encode for allothetic compass cues. Here, we apply a cosine response curve for coarse encoding of orientations. Such a mechanism was previously applied by other models (Haferlach et al., 2007; Kim and Lee, 2011). Neurons in the central complex of locusts contain a population-coded representation of allothetic compass cues based on the skylight polarization pattern (Heinze and Homberg, 2007). Similarly, central complex neurons in the *Drosophila* brain encode for heading orientations based on idiothetic self-motion and visual landmarks. Seelig and Jayaraman (2015) measured the fluorescent activity of genetically expressed calcium sensors indicating action potentials, while the fly was tethered on an air-suspended track ball system connected to a panoramic LED display. Any rotation of the fly on the ball is detected and fed back by corresponding motions of the visual scene on the display. The activity of 16 columnar neurons, which display the full circular range, generates a single maximum, which moves according to the turns of the fly on the ball. Interestingly, even though the representation is generated by visual stimuli, it can be accurately maintained solely by self-motion cues over the course of several seconds in the dark. A recent study on dung beetles (el Jundi et al., 2015), which navigate completely unaffected by landmarks, has shown that celestial compass cues are encoded in the central complex revealed by electrophysiological recordings. Taken together, it is likely that the central complex of social insects contains a similar neural coding of polarization- and landmark-based compass cues. Not only is the central complex function and anatomy highly conserved across insect species, but behavioral experiments on ants and bees also suggest the central role of using polarization and landmark cues for navigation. Our model further predicts allothetic goal-direction cues to be involved in PI mechanisms. Such neural representations have yet to be observed in experiments, ideally by applying the tethered track ball setup described in Seelig and Jayaraman (2015). A recent study has developed such a system for the use in desert ants (Dahmen et al., 2017), providing a powerful tool for future investigation of underlying neuronal mechanisms by combining this technology with electrophysiological recordings.

In our model, we assume that the agent's walking speed is neurally encoded as a linear signal that modulates the amplitude of HD activities by an additive gain. A similar, so-called gater mechanism has been applied in a model by Bernardet et al. (2008). Such linear speed signals have recently been found to be encoded by neurons in the rat's medial entorhinal cortex (Kropff et al., 2015) as well as the cockroach central complex (Martin et al., 2015). This shared encoding mechanism indicates the necessity of linear velocity components for accurate PI (Issa and Zhang, 2012). The temporal accumulation of speed-modulated HD signals in our model is achieved by a self-recurrent connection. Biologically, these recurrent connections can be interpreted as positive feedback within a group of neurons with the same preferred direction. Since our model applies PI as a scaffold for spatial learning, we apply this simplified accumulation mechanism to avoid random drifts observed in more complex attractor networks (Wang, 2001), which were applied in previous PI models (Touretzky et al., 1993; Hartmann and Wehner, 1995). We were also able to test the leaky-integrator hypothesis (Mittelstaedt and Glasauer, 1991) by fitting a single leakage parameter to observed behavioral data from desert ants (Müller and Wehner, 1988). The leakage parameter decreases the self-recurrent connection weight for leaky integration.

A HV representation is computed by using a cosine weight kernel, which was also used in Bernardet et al. (2008). Such a connectivity acts on each represented direction by adding the projections from other directions, respectively. This leads to the formation of an activity pattern with a single maximum across the population. The angle of the represented vector is readout by averaging the population vectors, while the distance is encoded by the amplitude of the population activity. We show that such a readout of a population-coded vector is sufficient to generate robust homing behavior in an artificial agent. Furthermore, it allows for accurate localization required for spatial learning of locations.

The extensive numerical analysis of noise affecting the accuracy of our PI mechanism leads to two predictions. First, PI accuracy seems to follow a similar function with respect to the noise levels for both the fully correlated and uncorrelated random fluctuations. While uncorrelated noise could be further filtered depending on the system size *N*, decorrelation of sensory input noise could be achieved by adding inhibitory feedback as shown in a model by Helias et al. (2014). Second, we varied the number of neurons *N* per layer for different levels of fully correlated noise, which predicts an accuracy plateau between 16 and 32 neurons where the accuracy will not increase for larger systems. This indicates that such a number of partitions for representing orientation variables is efficient and accurate enough. Interestingly, most prominent neuropils of the central complex exhibit a similar number of functional columns (Wolff et al., 2015). The central complex has been shown to be involved in sky compass processing (Heinze and Homberg, 2007), spatial orientation (Seelig and Jayaraman, 2015), and spatio-visual memory (Neuser et al., 2008; Ofstad et al., 2011). Its columnar and reverberating connectivity further supports the functional role of integrating orientation stimuli. These evidences suggest that the proposed circular arrays representing navigation vectors might be encoded in the central complex. We conclude that further experiments are needed to unravel how PI is exactly performed in the insect brain by closely linking neural activity and circuitry to behavioral function.

### 4.2. Reward-modulated vector acquisition and the role of motivational context

PI provides a possible mechanism for self-localization. As such, it has been shown experimentally that social insects apply this mechanism as a scaffold for spatial learning and memory (Collett et al., 2013). Here we propose a reward-modulated associative learning rule (Smith et al., 2008; Cassenaer and Laurent, 2012; Hige et al., 2015) for acquiring and storing vector representations. The acquisition and expression of such vector memories depend on the context during navigation. For GVs, the context is determined by the foraging state, which we model as a binary unit. Indeed, behavioral studies on desert (Wehner et al., 2006) and wood ants (Fernandes et al., 2015) have shown that expression of spatial memories is controlled by an internal state in a binary fashion. The association of the context with a reward signal, received at the feeder, drives synaptic weight changes corresponding to the difference between the current PI state and the respective weight. As this difference is minimized, the weights converge toward values representing the PI state when the reward was received at the feeder. Thus, like the HV, GVs are population-encoded with the angle determined by the position of the maximum activity and the length determined by the amplitude of the activity. To our knowledge, this is the first model that applies such a neural representation to perform vector-guided navigation. Previous models, such as Kubie and Fenton (2009); Cruse and Wehner (2011), do not provide possible underlying neural implementations of the PI-based stored information used for navigation. The HD accumulator model (Kubie and Fenton, 2009) argued that vector information is stored in so-called shortcut matrices, which are subsequently used for navigating toward goals. Similarly, the Cruse and Wehner model (Cruse and Wehner, 2011) stored HVs as geocentric coordinates in the activity of two neurons. Although it has been argued that this representation is biologically plausible, it is unlikely that persistent activity can explain global vector memories which are expressed over several days (Wehner et al., 2004). Furthermore, representing a two-dimensional variable requires at least three neurons, because firing rates are strictly positive. As such, existing models offer sufficient mechanisms in order to generate vector-guided navigation, they neither seem biologically plausible nor provide any explanations how such information is dynamically learned during navigation.

Our proposed encoding of GVs is validated by recent findings from a behavioral study on wood ants (Fernandes et al., 2015). The authors carried out a series of novel experimental paradigms involving training and testing channels. In the training channel, ants were trained to walk from their nest to a feeder at a certain distance, before they were transferred to the testing channel. There, they measured the expression of vector memories by observing the behavior. The authors showed that vector memories are expressed by successful association of direction and distance, therefore such memories might be encoded in a common neural population of the insect brain. The acquisition of vectors were rapid after 4–5 training trials, which corresponds to the rapid vector learning shown by our model during learning walks (Figure 8). However, the study mainly examined the expression of homeward vector memories which are not included in our model, because here the agent applies PI for homing. Recent work by Fleischmann et al. (2016) investigates landmark learning and memory during naturalistic foraging in the desert ant species *Cataglyphis fortis*. Like other desert ants, they spent the initial weeks of their lifetime inside the nest, before spending about a week foraging repeatedly for food to bring back to the nest. By placing controlled, prominent landmarks around the nest, the authors could measure the foraging routes of individual, marked ants. They also measured the accuracy of landmark-guided memories by transferring inward running ants right before they entered the nest. Their results show that ants initially forage only within a short distance and duration, but more experienced foragers increase their average foraging range and duration. Furthermore, they paths become straighter and they are more successful in finding food (also shown in another desert ant species; Wehner et al., 2004). Taken together, their results indicate that landmark learning and memory is a gradual process. Our model does not model landmark guidance during foraging, but it provides a simple strategy that could support this gradual learning mechanism. Specifically, it could provide the agent with a directional bias, by which the agent can learn visual routes toward rewarding food sources (Ardin et al., 2016). Finally, possible interactions between path integration and landmark-based memories has been recently shown in behavioral experiments (Wystrach et al., 2015), and as such, a complete neural model of naturalistic foraging behavior remains to be future work.

Two major higher brain areas in social insects exhibit experience-dependent plasticity due to foraging activity: the mushroom bodies (Yilmaz et al., 2016) and the central complex (Schmitt et al., 2016). The mushroom bodies are paired neuropils known to be involved in olfactory learning and memory (Owald and Waddell, 2015), as well as visual learning in discrimination tasks (Vogt et al., 2014). Studies on the central complex across various insect species have revealed its role in visual object localization (Seelig and Jayaraman, 2013) and visual learning (Liu et al., 2006), motor adaptation (Strauss, 2002), spatio-visual memory (Neuser et al., 2008; Seelig and Jayaraman, 2015; Ofstad et al., 2011), as well as polarization-based compass (Heinze and Homberg, 2007). A common coding principle in the central complex appears to be the topological mapping of stimuli within the full azimuthal circle (Plath and Barron, 2015). Both higher brain neuropils involve the functional diversity of multiple neuropeptides and neurotransmitters (Kahsai et al., 2012). The short neuropeptide F is a likely candidate influencing the foraging state, as it has been shown to regulate feeding behavior and foraging activity after starvation (Kahsai et al., 2010). Based on this evidence, we conclude that the population-coded vector memories described by our model are likely to be found in the central complex. Nonetheless, we do not exclude the possibility of possible interactions between the central complex and the mushroom bodies involved in spatial learning and navigation, which is supported by recent findings on novelty choice behavior in *Drosophila* (Solanki et al., 2015).

We proposed a novel computational model for PI and the acquisition and expression of vector memories in artificial agents. Although existing vertebrate and invertebrate models (Kubie and Fenton, 2009; Cruse and Wehner, 2011) have followed a similar approach of implementing vector-guided navigation, here we provide plausible neural implementations of the underlying control and learning mechanisms. Tested on a simulated agent, we show that the proposed model produces navigational behavior in the context of realistic closed-loop body-environment interactions (Webb, 1995; Seth et al., 2005; Pfeifer et al., 2007). In our previous work, we applied this approach to study adaptive locomotion and climbing (Manoonpong et al., 2013; Goldschmidt et al., 2014; Manoonpong et al., 2014), goal-directed behavior (Dasgupta et al., 2014) and memory-guided decision-making (Dasgupta et al., 2013). Although our model does not reproduce the full repertoire of insect navigation, it has shown to be sufficient in generating robust and efficient vector-guided navigation. Besides behavioral observations, our model also provides predictions about the structure and plasticity of related neural circuits in the insect brain (Haberkern and Jayaraman, 2016). We discussed our findings in the context of neurobiological evidences related to two higher brain areas of insects, the central complex and the mushroom bodies. We therefore conclude that our model offers a novel computational model for studying vector-guided navigation in social insects, which combines neural mechanisms with their generated behaviors. This can guide future behavioral and neurobiological experiments needed to evaluate our findings.

## Author contributions

Conceived and designed the experiments: DG, SD, and PM. Performed the experiments: DG. Analyzed the data: DG, SD, and PM. Contributed reagents/materials/analysis tools: DG and SD. Wrote the paper: DG, SD, and PM.

## Funding

This research was supported by Centre for BioRobotics (CBR) at University of Southern Denmark (SDU, Denmark). DG was supported by the Fundação para a Ciência e Tecnologia (FCT). PM was supported by Bernstein Center for Computational Neuroscience II Göttingen (BCCN grant 01GQ1005A, project D1) and Horizon 2020 Framework Programme (FETPROACT-01-2016—FET Proactive: emerging themes and communities) under grant agreement no. 732266 (Plan4Act). The funders had no role in study design, data collection and analysis, decision to publish, or preparation of the manuscript.

### Conflict of interest statement

The authors declare that the research was conducted in the absence of any commercial or financial relationships that could be construed as a potential conflict of interest.

## Supplementary material

Supplementary Video **Path integration and global vector learning in a simulated hexapod robot**.

## References

[B1] Ardin P. Peng F. Mangan M. Lagogiannis K. Webb B. (2016). Using an insect mushroom body circuit to encode route memory in complex natural environments. PLoS Comput. Biol. 12:e1004683. 10.1371/journal.pcbi.1004683 26866692 PMC4750948

[B2] Arena P. Patanè L. Termini P. S. (2014). A Computational Model for the Insect Brain. Cham: Springer International Publishing.

[B3] Averbeck B. B. Latham P. E. Pouget A. (2006). Neural correlations, population coding and computation. Nat. Rev. Neurosci. 7, 358–366. 10.1038/nrn1888 16760916

[B4] Benhamou S. Sauvé J.-P. Bovet P. (1990). Spatial memory in large scale movements: efficiency and limitation of the egocentric coding process. J. Theor. Biol. 145, 1–12. 10.1016/S0022-5193(05)80531-4

[B5] Bernardet U. Bermúdez i Badia S. Verschure P. F. M. J. (2008). A model for the neuronal substrate of dead reckoning and memory in arthropods: a comparative computational and behavioral study. Theor. Biosci. 127, 163–175. 10.1007/s12064-008-0038-8 18427853

[B6] Bienenstock E. Cooper L. Munro P. (1982). Theory for the development of neuron selectivity: orientation specificity and binocular interaction in visual cortex. J. Neurosci. 2, 32–48. 7054394 10.1523/JNEUROSCI.02-01-00032.1982 PMC6564292

[B7] Biesmeijer J. C. de Vries H. (2001). Exploration and exploitation of food sources by social insect colonies: a revision of the scout-recruit concept. Behav. Ecol. Sociobiol. 49, 89–99. 10.1007/s002650000289

[B8] Bolek S. Wolf H. (2015). Food searches and guiding structures in north african desert ants, cataglyphis. J. Comp. Physiol. A 201, 631–644. 10.1007/s00359-015-0985-8 25663433 PMC4439442

[B9] Bovet P. Benhamou S. (1988). Spatial analysis of animals' movements using a correlated random walk model. J. Theor. Biol. 131, 419–433. 10.1016/S0022-5193(88)80038-9

[B10] Buehlmann C. Graham P. Hansson B. S. Knaden M. (2014). Desert ants locate food by combining high sensitivity to food odors with extensive crosswind runs. Curr. Biol. 24, 960–964. 10.1016/j.cub.2014.02.056 24726153

[B11] Bühlmann C. Cheng K. Wehner R. (2011). Vector-based and landmark-guided navigation in desert ants inhabiting landmark-free and landmark-rich environments. J. Exp. Biol. 214, 2845–2853. 10.1242/jeb.054601 21832127

[B12] Burak Y. Fiete I. R. (2009). Accurate path integration in continuous attractor network models of grid cells. PLoS Comput. Biol. 5:e1000291. 10.1371/journal.pcbi.1000291 19229307 PMC2632741

[B13] Capaldi E. A. Smith A. D. Osborne J. L. Fahrbach S. E. Farris S. M. Reynolds D. R.. (2000). Ontogeny of orientation flight in the honeybee revealed by harmonic radar. Nature 403, 537–540. 10.1038/35000564 10676960

[B14] Cassenaer S. Laurent G. (2012). Conditional modulation of spike-timing-dependent plasticity for olfactory learning. Nature 482, 47–52. 10.1038/nature10776 22278062

[B15] Cheng K. Schultheiss P. Schwarz S. Wystrach A. Wehner R. (2014). Beginnings of a synthetic approach to desert ant navigation. Behav. Process. 102, 51–61. 10.1016/j.beproc.2013.10.001 24129029

[B16] Cheung A. (2014). Animal path integration: a model of positional uncertainty along tortuous paths. J. Theor. Biol. 341, 17–33. 10.1016/j.jtbi.2013.09.031 24096099

[B17] Cheung A. Vickerstaff R. (2010). Finding the way with a noisy brain. PLoS Comput. Biol. 6:e1000992. 10.1371/journal.pcbi.1000992 21085678 PMC2978673

[B18] Collett M. (2012). How navigational guidance systems are combined in a desert ant. Curr. Biol. 22, 927–932. 10.1016/j.cub.2012.03.049 22521785

[B19] Collett M. Cardé R. T. (2014). Navigation: many senses make efficient foraging paths. Curr. Biol. 24, R362–R364. 10.1016/j.cub.2014.04.001 24801185

[B20] Collett M. Chittka L. Collett T. S. (2013). Spatial memory in insect navigation. Curr. Biol. 23, R789–R800. 10.1016/j.cub.2013.07.020 24028962

[B21] Collett M. Collett T. S. (2009). The learning and maintenance of local vectors in desert ant navigation. J. Exp. Biol. 212, 895–900. 10.1242/jeb.024521 19282485

[B22] Collett M. Collett T. S. Bisch S. Wehner R. (1998). Local and global vectors in desert ant navigation. Nature 394, 269–272.

[B23] Collett M. Collett T. S. Wehner R. (1999). Calibration of vector navigation in desert ants. Curr. Biol. 9, 1031–1034. 10.1016/s0960-9822(99)80451-5 10508615

[B24] Collett T. Collett M. (2015). Route-segment odometry and its interactions with global path-integration. J. Comp. Physiol. A 201, 617–630. 10.1007/s00359-015-1001-z 25904159

[B25] Collett T. Collett M. Wehner R. (2001). The guidance of desert ants by extended landmarks. J. Exp. Biol. 204, 1635–1639. 10.5167/uzh-690 11398752

[B26] Cruse H. Wehner R. (2011). No need for a cognitive map: decentralized memory for insect navigation. PLoS Comput. Biol. 7:e1002009. 10.1371/journal.pcbi.1002009 21445233 PMC3060166

[B27] Dahmen H. Wahl V. L. Pfeffer S. E. Mallot H. A. Wittlinger M. (2017). Naturalistic path integration of cataglyphis desert ants on an air-cushioned lightweight spherical treadmill. J. Exp. Biol. 220, 634–644. 10.1242/jeb.148213 28202651

[B28] Dasgupta S. Wörgötter F. Manoonpong P. (2013). Information dynamics based self-adaptive reservoir for delay temporal memory tasks. Evolv. Syst. 4, 235–249. 10.1007/s12530-013-9080-y

[B29] Dasgupta S. Wörgötter F. Manoonpong P. (2014). Neuromodulatory adaptive combination of correlation-based learning in cerebellum and reward-based learning in basal ganglia for goal-directed behavior control. Front. Neural Circ. 8:126. 10.3389/fncir.2014.00126 25389391 PMC4211401

[B30] De Marco R. Menzel R. (2005). Encoding spatial information in the waggle dance. J. Exp. Biol. 208, 3885–3894. 10.1242/jeb.01832 16215216

[B31] Dubnau J. Chiang A.-S. (2013). Systems memory consolidation in drosophila. Curr. Opin. Neurobiol. 23, 84–91. 10.1016/j.conb.2012.09.006 23084099

[B32] Duer A. Paffhausen B. H. Menzel R. (2015). High order neural correlates of social behavior in the honeybee brain. J. Neurosci. Methods 254, 1–9. 10.1016/j.jneumeth.2015.07.004 26192327

[B33] el Jundi B. Homberg U. (2012). Receptive field properties and intensity-response functions of polarization-sensitive neurons of the optic tubercle in gregarious and solitarious locusts. J. Neurophysiol. 108, 1695–1710. 10.1152/jn.01023.2011 22773775

[B34] el Jundi B. Warrant E. J. Byrne M. J. Khaldy L. Baird E. Smolka J.. (2015). Neural coding underlying the cue preference for celestial orientation. Proc. Natl. Acad. Sci. U.S.A. 112, 11395–11400. 10.1073/pnas.1501272112 26305929 PMC4568659

[B35] Etienne A. S. Jeffery K. J. (2004). Path integration in mammals. Hippocampus 14, 180–192. 10.1002/hipo.10173 15098724

[B36] Eurich C. W. Schwegler H. (1997). Coarse coding: calculation of the resolution achieved by a population of large receptive field neurons. Biol. Cybernet. 76, 357–363. 10.1007/s004220050349 9237361

[B37] Evangelista C. Kraft P. Dacke M. Labhart T. Srinivasan M. V. (2014). Honeybee navigation: critically examining the role of the polarization compass. Philos. Trans. R. Soc. Lond. B. Biol. Sci. 369:20130037. 10.1098/rstb.2013.0037 24395964 PMC3886325

[B38] Fernandes A. S. D. Philippides A. Collett T. S. Niven J. E. (2015). The acquisition and expression of memories of distance and direction in navigating wood ants. J. Exp. Biol. 218, 3580–3588. 10.1242/jeb.125443 26417013

[B39] Fleischmann P. N. Christian M. Müller V. L. Rössler W. Wehner R. (2016). Ontogeny of learning walks and the acquisition of landmark information in desert ants, *Cataglyphis fortis*. J. Exp. Biol. 219, 3137–3145. 10.1242/jeb.140459 27481270

[B40] Friedrich R. W. Stopfer M. (2001). Recent dynamics in olfactory population coding. Curr. Opin. Neurobiol. 11, 468–474. 10.1016/S0959-4388(00)00236-1 11502394

[B41] Gaussier P. Joulain C. Banquet J. P. Leprêtre S. Revel A. (2000). The visual homing problem: an example of robotics/biology cross fertilization. Robot. Auton. Syst. 30, 155–180. 10.1016/S0921-8890(99)00070-6

[B42] Goldschmidt D. Wörgötter F. Manoonpong P. (2014). Biologically-inspired adaptive obstacle negotiation behavior of hexapod robots. Front. Neurorobot. 8:3. 10.3389/fnbot.2014.00003 24523694 PMC3905219

[B43] Graham P. Cheng K. (2009). Ants use the panoramic skyline as a visual cue during navigation. Curr. Biol. 19, R935–R937. 10.1016/j.cub.2009.08.015 19889365

[B44] Haberkern H. Jayaraman V. (2016). Studying small brains to understand the building blocks of cognition. Curr. Opin. Neurobiol. 37, 59–65. 10.1016/j.conb.2016.01.007 26826948

[B45] Haferlach T. Wessnitzer J. Mangan M. Webb B. (2007). Evolving a neural model of insect path integration. Adapt. Behav. 15, 273–287. 10.1177/1059712307082080

[B46] Hartmann G. Wehner R. (1995). The ant's path integration system: a neural architecture. Biol. Cybernet. 73, 483–497. 10.1007/bf00199541

[B47] Heinze S. Gotthardt S. Homberg U. (2009). Transformation of polarized light information in the central complex of the locust. J. Neurosci. 29, 11783–11793. 10.1523/JNEUROSCI.1870-09.2009 19776265 PMC6666666

[B48] Heinze S. Homberg U. (2007). Maplike representation of celestial e-vector orientations in the brain of an insect. Science 315, 995–997. 10.1126/science.1135531 17303756

[B49] Heinze S. Homberg U. (2009). Linking the input to the output: new sets of neurons complement the polarization vision network in the locust central complex. J. Neurosci. 29, 4911–4921. 10.1523/JNEUROSCI.0332-09.2009 19369560 PMC6665345

[B50] Helias M. Tetzlaff T. Diesmann M. (2014). The correlation structure of local neuronal networks intrinsically results from recurrent dynamics. PLoS Comput. Biol. 10:e1003428. 10.1371/journal.pcbi.1003428 24453955 PMC3894226

[B51] Hige T. Aso Y. Modi M. N. Rubin G. M. Turner G. C. (2015). Heterosynaptic plasticity underlies aversive olfactory learning in drosophila. Neuron 88, 985–998. 10.1016/j.neuron.2015.11.003 26637800 PMC4674068

[B52] Hoinville T. Wehner R. Cruse H. (2012). Learning and retrieval of memory elements in a navigation task, in Biomimetic and Biohybrid Systems, Vol. 7375, *Lecture Notes in Computer Science*, eds Prescott T. Lepora N. Mura A. Verschurepages P. (Berlin; Heidelberg: Springer), 120–131.

[B53] Homberg U. Heinze S. Pfeiffer K. Kinoshita M. el Jundi B. (2011). Central neural coding of sky polarization in insects. Philoso. Trans. R. Soc. Lond. B Biol. Sci. 366, 680–687. 10.1098/rstb.2010.0199 21282171 PMC3049008

[B54] Issa J. B. Zhang K. (2012). Universal conditions for exact path integration in neural systems. Proc. Natl. Acad. Sci. U.S.A. 109, 6716–6720. 10.1073/pnas.1119880109 22493275 PMC3340063

[B55] Jauffret A. Cuperlier N. Gaussier P. (2015). From grid cells and visual place cells to multimodal place cell: a new robotic architecture. Front. Neurorobot. 9:1. 10.3389/fnbot.2015.00001 25904862 PMC4388131

[B56] Kahsai L. Carlsson M. Winther Å. Nässel D. (2012). Distribution of metabotropic receptors of serotonin, dopamine, gaba, glutamate, and short neuropeptide f in the central complex of drosophila. Neuroscience 208, 11–26. 10.1016/j.neuroscience.2012.02.007 22361394

[B57] Kahsai L. Martin J.-R. Winther Å. M. E. (2010). Neuropeptides in the drosophila central complex in modulation of locomotor behavior. J. Exp. Biol. 213, 2256–2265. 10.1242/jeb.043190 20543124

[B58] Kim D. Lee J. (2011). Path integration mechanism with coarse coding of neurons. Neural Process. Lett. 34, 277–291. 10.1007/s11063-011-9198-5

[B59] Kropff E. Carmichael J. E. Moser M.-B. Moser E. I. (2015). Speed cells in the medial entorhinal cortex. Nature. 523, 419–424 10.1038/nature14622 26176924

[B60] Kubie J. L. Fenton A. A. (2009). Heading-vector navigation based on head-direction cells and path integration. Hippocampus 19, 456–479. 10.1002/hipo.20532 19072761

[B61] Lambrinos D. Kobayashi H. Pfeifer R. Maris M. Labhart T. Wehner R. (1997). An autonomous agent navigating with a polarized light compass. Adapt. Behav. 6, 131–161. 10.1177/105971239700600104

[B62] Lambrinos D. Möller R. Labhart T. Pfeifer R. Wehner R. (2000). A mobile robot employing insect strategies for navigation. Robot. Auton. Syst. 30, 39–64. 10.1016/S0921-8890(99)00064-0

[B63] Lebhardt F. Koch J. Ronacher B. (2012). The polarization compass dominates over idiothetic cues in path integration of desert ants. J. Exp. Biol. 215, 526–535. 10.1242/jeb.060475 22246261

[B64] Liu G. Seiler H. Wen A. Zars T. Ito K. Wolf R.. (2006). Distinct memory traces for two visual features in the drosophila brain. Nature 439, 551–556. 10.1038/nature04381 16452971

[B65] Madl T. Chen K. Montaldi D. Trappl R. (2015). Computational cognitive models of spatial memory in navigation space: a review. Neural Netw. 65, 18–43. 10.1016/j.neunet.2015.01.002 25659941

[B66] Mangan M. Webb B. (2012). Spontaneous formation of multiple routes in individual desert ants (*Cataglyphis velox*). Behav. Ecol. 23, 944–954. 10.1093/beheco/ars051

[B67] Manoonpong P. Dasgupta S. Goldschmidt D. Wörgötter F. (2014). Reservoir-based online adaptive forward models with neural control for complex locomotion in a hexapod robot, in 2014 International Joint Conference on Neural Networks (IJCNN) (Beijing), 3295–3302.

[B68] Manoonpong P. Parlitz U. Wörgötter F. (2013). Neural control and adaptive neural forward models for insect-like, energy-efficient, and adaptable locomotion of walking machines. Front. Neural Circ. 7:12. 10.3389/fncir.2013.00012 23408775 PMC3570936

[B69] Martin J. P. Guo P. Mu L. Harley C. M. Ritzmann R. E. (2015). Central-complex control of movement in the freely walking cockroach. Curr. Biol. 25, 2795–2803. 10.1016/j.cub.2015.09.044 26592340

[B70] Mathews Z. Lechón M. Calvo J. Dhir A. Duff A. Bermúdez i Badia S. (2009). Insect-like mapless navigation based on head direction cells and contextual learning using chemo-visual sensors, in IEEE/RSJ International Conference on Intelligent Robots and Systems, 2009 (St. Louis, MO), 2243–2250.

[B71] Menzel R. Greggers U. Smith A. Berger S. Brandt R. Brunke S.. (2005). Honey bees navigate according to a map-like spatial memory. Proc. Natl. Acad. Sci. U.S.A. 102, 3040–3045. 10.1073/pnas.0408550102 15710880 PMC549458

[B72] Merkle T. Knaden M. Wehner R. (2006). Uncertainty about nest position influences systematic search strategies in desert ants. J. Exp. Biol. 209, 3545–3549. 10.1242/jeb.02395 16943494

[B73] Mittelstaedt M.-L. Glasauer S. (1991). Idiothetic navigation in gerbils and humans. Zoologis. Jahrbuch Physiol. 95, 427–435.

[B74] Müller M. Wehner R. (1988). Path integration in desert ants, cataglyphis fortis. Proc. Natl. Acad. Sci. U.S.A. 85, 5287–5290. 10.1073/pnas.85.14.5287 16593958 PMC281735

[B75] Muser B. Sommer S. Wolf H. Wehner R. (2005). Foraging ecology of the thermophilic australian desert ant, melophorus bagoti. Aust. J. Zool. 53, 301–311. 10.1071/ZO05023

[B76] Narendra A. Gourmaud S. Zeil J. (2013). Mapping the navigational knowledge of individually foraging ants, myrmecia croslandi. Proc. R. Soc. Lond. B Biol. Sci. 280:20130683. 10.1098/rspb.2013.0683 23804615 PMC3712440

[B77] Neuser K. Triphan T. Mronz M. Poeck B. Strauss R. (2008). Analysis of a spatial orientation memory in drosophila. Nature 453, 1244–1247. 10.1038/nature07003 18509336

[B78] Ofstad T. A. Zuker C. S. Reiser M. B. (2011). Visual place learning in drosophila melanogaster. Nature 474, 204–207. 10.1038/nature10131 21654803 PMC3169673

[B79] Oja E. (1982). Simplified neuron model as a principal component analyzer. J. Math. Biol. 15, 267–273. 10.1007/BF00275687 7153672

[B80] Olshausen B. A. Field D. J. (1997). Sparse coding with an overcomplete basis set: a strategy employed by v1? Vis. Res. 37, 3311–3325. 9425546 10.1016/s0042-6989(97)00169-7

[B81] Owald D. Waddell S. (2015). Olfactory learning skews mushroom body output pathways to steer behavioral choice in drosophila. Curr. Opin. Neurobiol. 35, 178–184. 10.1016/j.conb.2015.10.002 26496148 PMC4835525

[B82] Pfeffer S. Bolek S. Wolf H. Wittlinger M. (2015). Nest and food search behaviour in desert ants, cataglyphis: a critical comparison. Anim. Cogn. 18, 885–894. 10.1007/s10071-015-0858-0 25805650

[B83] Pfeifer R. Lungarella M. Iida F. (2007). Self-organization, embodiment, and biologically inspired robotics. Science 318, 1088–1093. 10.1126/science.1145803 18006736

[B84] Plath J. A. Barron A. B. (2015). Current progress in understanding the functions of the insect central complex. Curr. Opin. Insect Sci. 12, 11–18. 10.1016/j.cois.2015.08.005

[B85] Salinas E. Abbott L. (1995). Transfer of coded information from sensory to motor networks. J. Neurosci. 15, 6461–6474. 7472409 10.1523/JNEUROSCI.15-10-06461.1995 PMC6578023

[B86] Schmid-Hempel P. (1984). Individually different foraging methods in the desert ant cataglyphis bicolor (hymenoptera, formicidae). Behav. Ecol. Sociobiol. 14, 263–271. 10.1007/BF00299497

[B87] Schmitt F. Stieb S. M. Wehner R. Rössler W. (2016). Experience-related reorganization of giant synapses in the lateral complex: potential role in plasticity of the sky-compass pathway in the desert ant *Cataglyphis fortis*. Dev. Neurobiol. 76, 390–404. 10.1002/dneu.22322 26138802

[B88] Schmolke A. Mallot H. Neurowissenschaft K. (2002). Polarization compass for robot navigation, in The Fifth German Workshop on Artificial Life (Lübeck), 163–167.

[B89] Seelig J. D. Jayaraman V. (2013). Feature detection and orientation tuning in the *Drosophila* central complex. Nature 503, 262–266. 10.1038/nature12601 24107996 PMC3830704

[B90] Seelig J. D. Jayaraman V. (2015). Neural dynamics for landmark orientation and angular path integration. Nature 521, 186–191. 10.1038/nature14446 25971509 PMC4704792

[B91] Seth A. K. Sporns O. Krichmar J. L. (2005). Neurorobotic models in neuroscience and neuroinformatics. Neuroinformatics 3, 167–170. 10.1385/NI:3:3:167 16077157

[B92] Smith D. Wessnitzer J. Webb B. (2008). A model of associative learning in the mushroom body. Biol. Cybernet. 99, 89–103. 10.1007/s00422-008-0241-1 18607623

[B93] Solanki N. Wolf R. Heisenberg M. (2015). Central complex and mushroom bodies mediate novelty choice behavior in drosophila. J. Neurogenet. 29, 30–37. 10.3109/01677063.2014.1002661 25585638

[B94] Srinivasan M. (2015). Where paths meet and cross: navigation by path integration in the desert ant and the honeybee. J. Comp. Physiol. A 201, 533–546. 10.1007/s00359-015-1000-0 25971358

[B95] Stein R. B. Gossen E. R. Jones K. E. (2005). Neuronal variability: noise or part of the signal? Nat. Rev. Neurosci. 6, 389–397. 10.1038/nrn1668 15861181

[B96] Strauss R. (2002). The central complex and the genetic dissection of locomotor behaviour. Curr. Opin. Neurobiol. 12, 633–638. 10.1016/S0959-4388(02)00385-9 12490252

[B97] Taube J. Muller R. Ranck J. (1990). Head-direction cells recorded from the postsubiculum in freely moving rats. I. description and quantitative analysis. J. Neurosci. 10, 420–435. 2303851 10.1523/JNEUROSCI.10-02-00420.1990 PMC6570151

[B98] Todorov E. Jordan M. I. (2002). Optimal feedback control as a theory of motor coordination. Nat. Neurosci. 5, 1226–1235. 10.1038/nn963 12404008

[B99] Touretzky D. Redish A. Wan H. (1993). Neural representation of space using sinusoidal arrays. Neural Comput. 5, 869–884. 10.1162/neco.1993.5.6.869

[B100] Vickerstaff R. J. (2007). Evolving Dynamical System Models of Path Integration. Ph.D. thesis, University of Sussex.

[B101] Vickerstaff R. J. Cheung A. (2010). Which coordinate system for modelling path integration? J. Theor. Biol. 263, 242–261. 10.1016/j.jtbi.2009.11.021 19962387

[B102] Vogt K. Schnaitmann C. Dylla K. V. Knapek S. Aso Y. Rubin G. M.. (2014). Shared mushroom body circuits underlie visual and olfactory memories in Drosophila. eLife 3:e02395. 10.7554/eLife.02395 25139953 PMC4135349

[B103] Wang X.-J. (2001). Synaptic reverberation underlying mnemonic persistent activity. Trends Neurosci. 24, 455–463. 10.1016/S0166-2236(00)01868-3 11476885

[B104] Webb B. (1995). Moving the frontiers between robotics and biology using robots to model animals: a cricket test. Robot. Auton. Syst. 16, 117–134. 10.1016/0921-8890(95)00044-5

[B105] Wehner R. (2003). Desert ant navigation: how miniature brains solve complex tasks. J. Comp. Physiol. A 189, 579–588. 10.1007/s00359-003-0431-1 12879352

[B106] Wehner R. Boyer M. Loertscher F. Sommer S. Menzi U. (2006). Ant navigation: one-way routes rather than maps. Curr. Biol. 16, 75–79. 10.1016/j.cub.2005.11.035 16401425

[B107] Wehner R. Meier C. Zollikofer C. (2004). The ontogeny of foraging behaviour in desert ants, cataglyphis bicolor. Ecol. Entomol. 29, 240–250. 10.1111/j.0307-6946.2004.00591.x

[B108] Weir P. T. Dickinson M. H. (2015). Functional divisions for visual processing in the central brain of flying *Drosophila*. Proce. Natl. Acad. Sci. U.S.A. 112, E5523–E5532. 10.1073/pnas.1514415112 26324910 PMC4603480

[B109] Wittlinger M. Wehner R. Wolf H. (2006). The ant odometer: stepping on stilts and stumps. Science 312, 1965–1967. 10.1126/science.1126912 16809544

[B110] Wittlinger M. Wehner R. Wolf H. (2007). The desert ant odometer: a stride integrator that accounts for stride length and walking speed. J. Exp. Biol. 210, 198–207. 10.1242/jeb.02657 17210957

[B111] Wittmann T. Schwegler H. (1995). Path integration – a network model. Biol. Cybernet. 73, 569–575. 10.1007/BF00199549

[B112] Wolf H. Wittlinger M. Bolek S. (2012). Re-visiting of plentiful food sources and food search strategies in desert ants. Front. Neurosci. 6:102. 10.3389/fnins.2012.00102 22783163 PMC3389614

[B113] Wolff T. Iyer N. A. Rubin G. M. (2015). Neuroarchitecture and neuroanatomy of the drosophila central complex: a gal4-based dissection of protocerebral bridge neurons and circuits. J. Comp. Neurol. 523, 997–1037. 10.1002/cne.23705 25380328 PMC4407839

[B114] Wystrach A. Dewar A. D. Graham P. (2014). Insect vision: emergence of pattern recognition from coarse encoding. Curr. Biol. 24, R78–R80. 10.1016/j.cub.2013.11.054 24456981

[B115] Wystrach A. Mangan M. Webb B. (2015). Optimal cue integration in ants. Proc. R. Soc. Lond. B Biol. Sci. 282:20151484. 10.1098/rspb.2015.1484 26400741 PMC4614770

[B116] Yilmaz A. Lindenberg A. Albert S. Grübel K. Spaethe J. Rössler W.. (2016). Age-related and light-induced plasticity in opsin gene expression and in primary and secondary visual centers of the nectar-feeding ant *Camponotus rufipes*. Dev. Neurobiol. 76, 1041–1057. 10.1002/dneu.22374 26724470

