# Supplementary material for: A Neurocomputational Model of Goal-Directed Navigation in Insect-Inspired Artificial Agents
Source: Front Neurorobot. 2017 Apr 12;11:20. doi: 10.3389/fnbot.2017.00020 (PMC5388780; doi:10.3389/fnbot.2017.00020)
Supplement: Supplementary file 2 [file Presentation1.pdf]

Supplementary Material: A neurocomputational model of goal-directed navigation in insect-inspired embodied agents

Dennis Goldschmidt [1,2,*], **Poramate Manoonpong** [3] **and Sakyasingha Dasgupta** [4,5]

*Correspondence:
Dennis Goldschmidt
Champalimaud Centre for the Unknown
Av Brasília, Doca de Pedrouços, 1400-038 Lisbon, Portugal
dennis.goldschmidt@neuro.fchampalimaud.org

1 SUPPLEMENTARY TABLES

1.1 Model variables & parameters

Symbol	Description	Value (range)
$\phi(t)$	compass orientation of the agent	$[0, 2\pi)$
$s(t)$	walking speed signal of the agent	$[0, 1]$
i	index of (postsynaptic) neurons in circular arrays	$[0, N-1]$
j	index of (presynaptic) neurons in circular arrays	$[0, N-1]$
N	number of neurons in circular arrays	18
t	simulation time	$[0, T]$
Δt	simulation time interval	0.1 s
t_{forage}	duration of outward foraging until homing	1000 s
T	total duration of foraging trial	$\frac{3}{2}t_{home}$
ϕ_i	preferred orientations of circular arrays	$[0, 2\pi)$
$x_i^{HD}(t)$	activity of ith neuron in head direction layer	$\mathbb{R}_{\geq 0}$
$x_i^{G}(t)$	activity of ith neuron in gating layer	$\mathbb{R}_{\geq 0}$
δ_{ij}	Kronecker delta	
$x_i^{M}(t)$	activity of ith neuron in memory layer	$\mathbb{R}_{\geq 0}$
λ	memory leak parameter	0.0075
$x_i^{HV}(t)$	activity of ith neuron in home vector array	$\mathbb{R}_{\geq 0}$
$w_{ij}(t)$	weights of decoding layer	$\mathbb{R}_{\geq 0}$
$\theta_{HV}(t)$	home vector angle (vector average of x_i^{HV})	$[0, 2\pi)$
$l_{HV}(t)$	length of home vector	$\mathbb{R}_{\geq 0}$
$m_{HV}(t)$	motor signal of home vector angle	$\mathbb{R}_{\geq 0}$
$r(t)$	food reward at the feeder	$[0, 1]$
$d(t)$	distance of the agent to the feeder	$\mathbb{R}_{\geq 0}$
$\sigma(t)$	binary foraging state of the agent	$[0, 1]$

Symbol	Description	Value (range)
$x_i^{GV}(t)$	activity of ith neuron in global vector array	$\mathbb{R}_{\geq 0}$
$w_i^{GV}(t)$	weight to ith neuron in global vector array	$\mathbb{R}_{\geq 0}$
μ_{GV}	global vector learning rate	2
$\theta_{GV}(t)$	global vector angle (vector average of x_i^{GV})	$[0, 2\pi)$
l_{GV}	length of global vector	$\mathbb{R}_{\geq 0}$
$m_{GV}(t)$	motor signal of global vector angle	$\mathbb{R}_{\geq 0}$
$m_{\varepsilon}(t)$	output signal of random search (exploration)	$\mathbb{R}$
$\varepsilon(t)$	exploration rate	$[0, 1]$
$v(t)$	lowpass filtered signal of food reward	$\mathbb{R}_{\geq 0}$
γ	food reward discount factor	$\mathbb{R}_{\geq 0}$
$\beta(t)$	inverse temperature of exploration rate	$\mathbb{R}_{\geq 0}$
μ_{β}	global learning rate for β adaptation	10^{-6}
μ_v	reward-based learning rate for β adaptation	10^2
$\Sigma(t)$	control output for steering	$\mathbb{R}$
ϕ_{noisy}	noisy compass orientation	$[0, 2\pi)$
$\delta\phi$	shift of compass orientation due to noise	$[0, 2\pi)$
η_{sens}	sensory noise level	$[0, 2\pi)$
η_{neur}	neural noise level	$\mathbb{R}_{\geq 0}$
δx_i^{HD}	fluctuations in the head direction activity	$\mathbb{R}$
δr	position error	$\mathbb{R}_{\geq 0}$
L_{feed}	distance of the nest to the feeder	$\mathbb{R}_{\geq 0}$
θ_{feed}	angle of the nest to the feeder	$[0, 2\pi)$
$p(x, y)$	probability density function (box histogram)	$[0, 1]$

2 EXPERIMENTAL PLATFORMS & FORAGING STATISTICS

2.1 Experimental Platforms

For our simulation results, we applied two different experimental platforms: First, we embedded the closed-loop control into a two-dimensional simulated point agent (Fig. S1i) for large-scale numerical results. Secondly, we used a simulated, embodied agent based on the the hexapod walking robot *AMOS II* (Fig. S1ii, Manoonpong et al. (2013)). Both agents are able to perceive sensory input about compass direction, walking speed, and landmark detection, as well as food reward and internally generated signals (e.g., foraging state). These external and internal signals are fed into our model.

Our navigation model produces an output signal which controls the steering direction of the agent. The embodied agent applies a central pattern generator (CPG)-based neural locomotion control, which consists of modular neural networks generating a variety of periodic patterns and coordinating all leg joints. Thus, the agent is able to control a multitude of different, insect-like behavioral patterns. The resulting behaviors include omnidirectional walking and insect-like gaits (Manoonpong et al., 2013), which can be controlled manually or autonomously driven by exteroceptive sensors, such as a camera (Zenker et al., 2013), a laser scanner (Kesper et al., 2013), or infrared sensors (Goldschmidt et al., 2014). All neural networks in the CPG-based locomotion control are modeled using a discrete-time non-spiking neuron model with different activation functions (see Manoonpong et al. (2013) for details).

2.2 Agent motion dynamics and foraging statistics

In this subsection, we will derive agent trajectory dynamics and foraging statistics for modeling social insects. In both the point and embodied agent cases, the motion trajectory of the agent can be modeled using the current compass orientation $\phi(t)$ as well as the walking speed $v(t)$. The Cartesian coordinates x

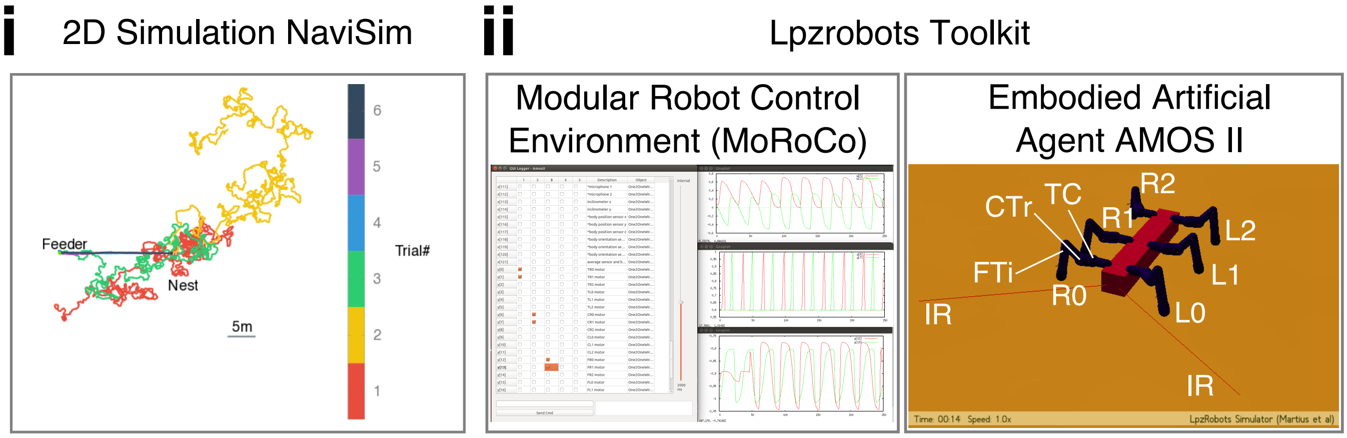

Figure S1. Experimental platforms. i) The twodimensional point agent simulation *NaviSim* is applied for large-scale numerical experiments. ii) *Lpzrobots* framework (Der and Martius, 2012) containing the Modular Robot Control Environment and the simulated artificial agent based on the six-legged walking robot *AMOS II* (Manoonpong et al., 2013). The agent has six legs (R0, R1, R2, L0, L1, L2) and each leg has three joints: the thoraco-coxal (TC) joint enables forward and backward movements, the coxa-trochanteral (CTr) joint enables elevation and depression of the leg, and the femur-tibia (FTi) joint enables extension and flexion of the tibia. The agent also contains a multitude of proprio- and exteroceptive sensors. Here we apply a compass sensor, a walking speed sensor, and infrared (IR) sensors. Both platforms are open-source projects and are available at https://github.com/degoldcode/NaviSim (NaviSim) and https://github.com/georgmartius/lpzrobots (Lpzrobots), respectively.

and y are described by the following differential equations

$$\dot{x}(t) = v(t)\cos(\phi(t)), \tag{S1}$$

$$\dot{y}(t) = v(t)\sin(\phi(t)) \tag{S2}$$

In the two-dimensional simulation, we numerically integrate these equations by using the forward Euler method with interval step size Δt as follows:

$$x(t + \Delta t) = x(t) + \Delta t \cdot \dot{x}(t), \tag{S3}$$

$$y(t + \Delta t) = y(t) + \Delta t \cdot \dot{y}(t). \tag{S4}$$

The orientation of the agent is updated given by the differential equation

$$\phi(t + \Delta t) = \phi(t) + \Delta t \cdot \dot{\phi}(t) = \phi(t) + \Delta t \cdot k_\phi \Sigma(t), \tag{S5}$$

where $\Sigma(t)$ is the control output of our model, and $k_\phi = \pi$ is a scaling factor. In random foraging, we apply a Gaussian normal distribution $\mathcal{N}(0, \exp(t))$ for the turning rate, which corresponds to a correlated random walk of the agent (Bovet and Benhamou, 1988). It has been shown that such a random walk leads to mean foraging distances $\langle L \rangle$ proportional to the square root of the simulation time $\sqrt{t}$.

Similarly, we can show that the path integration errors follow a similar square-root scaling law. Fig. S2 shows the position errors δr with respect to time for each of the 1000 trials. We averaged the position errors for each time step individually, which is indicated by the black colored solid line. These time-averaged position errors increase as a square root function of time (green-colored dashed line) with scaling factor $a = 0.0044$.

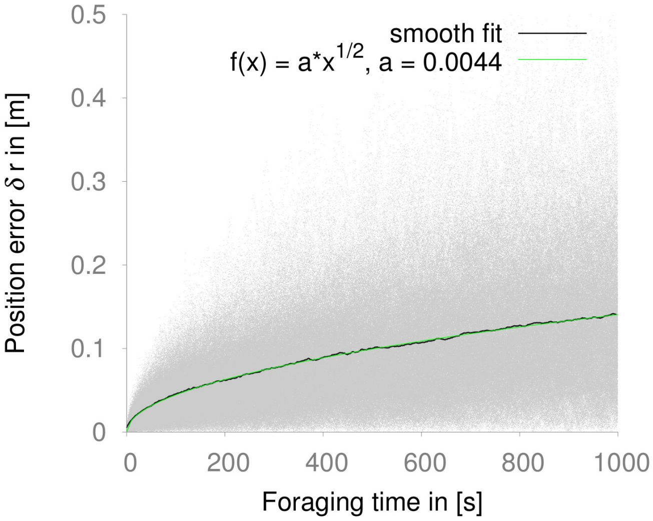

Figure S2. Position errors δr and mean errors $\langle \delta r \rangle_t$ for each time step with respect to foraging time in 1000 trials (fixed sensory noise of 5% and 18 neurons per layer). Smooth fit (black line) indicates an interpolation of the data points by taking the average of positional errors monotonically along the foraging time axis. We identified this function to scaled by the square root of the foraging time (green line).

3 DIRECTED WALK USING SINUSOIDAL PHASORS

3.1 Directed walk using sine error compensation

In order to generate directed motion of an agent towards a desired orientation, we apply a turning rate based on a sinusoidal function (Mittelstaedt, 1962, 1985; Vickerstaff and Di Paolo, 2005) minimizing the angular difference between the desired and the actual orientation of the agent. The angular difference, or error is defined as

$$\delta = \theta - \phi, \tag{S6}$$

$$\delta \in [-\pi, \pi) \tag{S7}$$

where θ is the desired orientation, and ϕ is the actual orientation. Thus, a turning rate given by

$$\dot{\phi} = k_\phi \sin(\delta) \tag{S8}$$

leads to right turns ($\dot{\phi} < 0$) when the actual orientation is left from the desired orientation ($\delta < 0$), and vice versa (see Fig. S3).

3.2 Generating searching patterns

An interesting behavior arises from the unstable fixed point given by Eq. S8. When the agent overshoots the home or goal position, its angular error changes rapidly from zero to close to the unstable fixed point $\pm\pi$. Note that if the agent's angular error is exactly $\pm\pi$, the turning rate is computed to be zero by definition. However, if δ is close to the unstable fixed point, the agent will slowly turn to the left or right, leading the turning rate to increase in the respective turning direction. As the agent's orientation aligns with the desired orientation, the turning rate decreases to zero. As a result, the agent will perform loops around the desired position in a searching pattern (Vickerstaff and Di Paolo, 2005). Indeed, such looped searching patterns

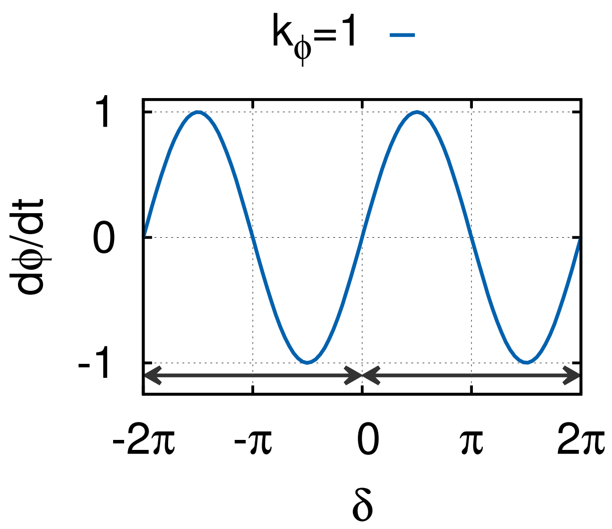

Figure S3. Sketch of the turning dynamics using a sine function of angular difference. The dynamical system consists of two fixed points: a stable one at $x = 0$ and an unstable one at $x = \pm\pi$. The system has been shown to be equivalent to a linearly damped pendulum (Vickerstaff, 2007).

has been observed in desert ants (Wehner and Srinivasan, 1981), as well as in honeybees (Reynolds et al., 2007).

3.3 Proof: phasor addition of inverted home vector and global vector leads to goal-directed phasor

Here, we prove that adding the phasors given by the inverted home vector (HV) and global vector (GV) leads to a phasor, which has a phase corresponding to the orientation towards the goal. We define the HV as vector **a** (angle θ_{HV}, length l_{HV}) and the GV as vector **b** (angle θ_{GV}, length l_{GV}). We will show that the vector $\mathbf{b} - \mathbf{a}$ connecting the agent's current position and the goal (see Fig. S4), is represented by the sum of the HV and GV phasors. We assume that the agent controls its heading orientation $\phi(t)$ due to the following differential control

$$\dot{\phi}(t) \propto l_{HV}(t)\sin(\theta_{HV}(t) - \phi(t) - \pi) + l_{GV}(t)\sin(\theta_{GV}(t) - \phi(t)), \qquad \text{(S9)}$$

where vector **a** is inversed by substraction of π. For convenience, we will drop the time dependences from now on.

Representing the phasors in the complex plane $\mathbb{C}$ with $c\sin(x) = \text{Im}\left[ce^{ix}\right]$ leads to

$$\begin{aligned}\dot{\phi} &\propto \text{Im}\left[l_{HV}e^{i\theta_{HV}}\underbrace{e^{-i\pi}}_{=-1}e^{-i\phi}\right] + \text{Im}\left[l_{GV}e^{i\theta_{GV}}e^{-i\phi}\right]\\ &= \text{Im}\left[(l_{GV}e^{i\theta_{GV}} - l_{HV}e^{i\theta_{HV}})e^{-i\phi}\right].\end{aligned}$$

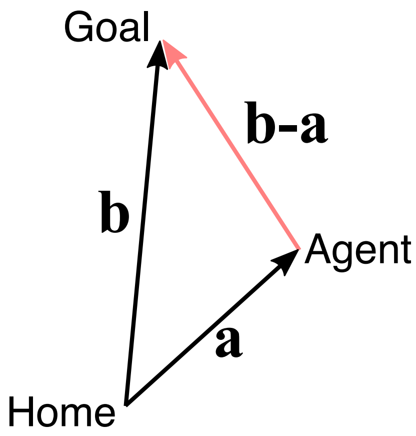

Figure S4. Sketch of vector-based navigation. In order to derive the correct orientation towards the goal based on a stored vector **b**, the agent has to subtract by the current position **a** derived from path integration.

Clearly, $(l_{GV}e^{i\theta_{GV}} - l_{HV}e^{i\theta_{HV}})$ is the vector $(\mathbf{b} - \mathbf{a})$ described in complex polar coordinates. Thus, we define the agent-to-goal vector representation in complex polar coordinates to be $l_{goal}e^{i\theta_{goal}}$ to obtain

$$\dot{\phi} \propto \text{Im}\left[l_{goal}e^{i\theta_{goal}}e^{-i\phi}\right] = l_{goal}\sin(\theta_{goal} - \phi)$$

Therefore, we proved that the addition of the inverted home vector and the global vector phasor leads to a phasor that describes the vector connecting the agent's current location to the goal position.

4 DERIVING AN ADAPTIVE EXPLORATION RATE BASED ON A GRADIENT RULE

Here, we derive an adaptive exploration rate based on a gradient rule (Triesch, 2005, 2007) for a foraging agent in random environments (i.e., containing randomly distributed goals). The exploration rate only accounts for received rewards in time. We define the time-discounted cumulative reward to be

$$v(t) = \sum_{i=0}^{t} \gamma^i r(t-i),$$

which is given by the update rule $v \leftarrow r + \gamma v$. We assume the exploration rate with respect to v to be given by

$$\varepsilon(v(t)) = \exp(-\beta v(t)),$$

where $\beta > 0$ is the inverse temperature. For later convenience, we derive the partial derivatives of ε, which are given by

$$\frac{\partial\varepsilon(v)}{\partial\beta} = -v\exp(-\beta v) = -v\varepsilon, \quad \text{(S10)}$$

$$\frac{\partial\varepsilon(v)}{\partial v} = -\beta\exp(-\beta v) = -\beta\varepsilon. \quad \text{(S11)}$$

We derive a gradient rule, which changes β accordingly to bring the probability distribution $f_\varepsilon(\varepsilon)$ of $\varepsilon(t)$ closer to an exponential distribution $f_{exp}(\lambda, \varepsilon) = \lambda \exp(-\lambda\varepsilon)$, assuming a fixed mean distribution. This maximizes the mutual information between the input distribution $f_v(v)$ and the output distribution$f_\varepsilon(\varepsilon)$, such that the exploration activity matches the environmental needs. The following relationship is given by the derivatives:

$$f_\varepsilon(\varepsilon) = \frac{f_v(v)}{\frac{\partial\varepsilon}{\partial v}}. \tag{S12}$$

We consider the Kullback-Leibler (KL) divergence as a measure for closeness of two probability distributions:

$$\begin{aligned} D_{KL}(f_\varepsilon|f_{\text{exp}}) &= \int f_\varepsilon(\varepsilon) \ln\left(\frac{f_\varepsilon(\varepsilon)}{\lambda\exp(-\lambda\varepsilon)}\right) d\varepsilon \\ &= \int f_\varepsilon(\varepsilon)\Big[\ln\big(f_\varepsilon(\varepsilon)\big) - \ln\big(\lambda\exp(-\lambda\varepsilon)\big)\Big] d\varepsilon \\ &= \int f_\varepsilon(\varepsilon)\ln(f_\varepsilon(\varepsilon))d\varepsilon - \int f_\varepsilon(\varepsilon)(\ln\lambda - \lambda\varepsilon)d\varepsilon \\ &= -H(\varepsilon) + \lambda\int \varepsilon f_\varepsilon(\varepsilon)d\varepsilon - \ln\lambda \int f_\varepsilon(\varepsilon)d\varepsilon \\ &= -H[\varepsilon] + \lambda E[\varepsilon] - \ln\lambda + \text{const.} \end{aligned}$$

By substituting $-H[\varepsilon]$ with the relations given by Eq. S12, we derive

$$\begin{aligned} -H[\varepsilon] &= \int f_\varepsilon(\varepsilon)\ln(f_\varepsilon(\varepsilon))d\varepsilon = \int f_v(v)\ln\left(\frac{f_v(v)}{\frac{\partial\varepsilon}{\partial v}}\right)dv = \int f_v(v)\left[\ln\big(f_v(v)\big) - \ln\left(\frac{\partial\varepsilon}{\partial v}\right)\right]dv \\ &= \int f_v(v)\ln\big(f_v(v)\big)dv - \int f_v(v)\ln\left(\frac{\partial\varepsilon}{\partial v}\right)dv = -H[v] - E\left[\ln\left(\frac{\partial\varepsilon}{\partial v}\right)\right], \end{aligned}$$

which leads to

$$D_{KL}(f_\varepsilon|f_{\text{exp}}) = -H[v] - E\left[\ln\left(\frac{\partial\varepsilon}{\partial v}\right)\right] + \lambda E[\varepsilon] - \ln\lambda + \text{const.} \tag{S13}$$

Considering that the input entropy $H[v]$ is independent[1] of β, we derive the partial derivative of the KL divergence with respect to β as given by

$$\begin{aligned} \frac{\partial D_{KL}}{\partial\beta} &= \frac{\partial}{\partial\beta}\left\{-E\left[\ln\left(\frac{\partial\varepsilon}{\partial v}\right)\right] + \lambda E[\epsilon]\right\} = -\frac{\partial}{\partial\beta}\left\{E\left[\ln\left(\frac{\partial\varepsilon}{\partial v}\right) - \lambda\epsilon\right]\right\} \\ &= -E\left[\frac{\partial\ln(\partial\varepsilon/\partial v)}{\partial\beta} - \lambda\frac{\partial\varepsilon}{\partial\beta}\right] = -E\left[\frac{\partial\ln(-\beta\varepsilon)}{\partial\beta} + \lambda v\varepsilon\right], \end{aligned}$$

[1] It follows $\frac{\partial H[v]}{\partial\beta} = 0$.

where we used the derivatives from Eqs. S10 and S11. Thus, the partial derivivate of the KL divergence is given by

$$\frac{\partial D_{KL}}{\partial \beta} = -E\left[\frac{1}{\beta} + \lambda v \varepsilon\right], \tag{S14}$$

where we used $\frac{f_V(V)}{f_\varepsilon(\epsilon)} = \frac{\partial \varepsilon}{\partial V}$.

Finally, the gradient-descent rule is given by

$$\Delta\beta(t) = \eta_\beta \left(\frac{1}{\beta(t)} + \lambda v(t)\varepsilon(t)\right), \tag{S15}$$

which we apply as an update for $\beta \leftarrow \beta + \Delta\beta$.

5 PSEUDOCODE OF LEARNING ALGORITHM FOR ADAPTIVE VECTOR NAVIGATION

Algorithm 1 Learning algorithm for adaptive vector navigation

Initialize: $w_i^{GV} = 0$, all activities x_i^{HV} and x_i^{GV} of the circular arrays are initially set to zero. See Supplementary Tables for detailed initial parameters.

Repeat: At simulation time t

Step 1: Update sensory inputs (compass $\phi(t)$, speed $s(t)$, internal states $\sigma(t)$ and rewards $R(t)$ from environmental interactions of the agent.

Step 2: Update home vector array activities $x_i^{\text{HV}}(t)$ using Eqs. 4–10.

Step 3: Update global vector array activities $x_i^{\text{GV}}(t)$ and weights $w_i^{\text{GV}}(t)$ using Eqs. 12–15.

Step 5: The exploration rate $\varepsilon(t)$ is updated using Eqs. 19 and 20.
$v \leftarrow r + \gamma v$
$\varepsilon \leftarrow \exp(-\beta v)$

Step 6: Update inverse temperature $\beta(t)$ of exploration rate using Eqs. 21 and 22.
$\Delta\beta \leftarrow \mu_\beta \left(\frac{1}{\beta} + \mu_v v \varepsilon\right)$
$\beta \leftarrow \beta + \Delta\beta,$

Step 7: Update navigation control output $\Sigma(t)$ as a weighted linear combination of expressed vector representations and exploration using Eq. 23.

Step 8: Update agent's position due to control output.
$\phi \leftarrow \phi + \Delta t \kappa_\phi \Sigma$
$\mathbf{r}_{\text{agent}} \leftarrow \mathbf{r}_{\text{agent}} + \Delta t \begin{pmatrix} v\cos(\phi) \\ v\sin(\phi) \end{pmatrix}$
Step 9: $t \leftarrow t + \Delta t$.

Until: maximum simulation time is reached ($t = T$).

REFERENCES

Bovet, P. and Benhamou, S. (1988). Spatial analysis of animals' movements using a correlated random walk model. *Journal of Theoretical Biology*, 131(4):419 – 433.

Der, R. and Martius, G. (2012). *The Playful Machine: Theoretical Foundation and Practical Realization of Self-Organizing Robots*, volume 15 of *Cognitive Systems Monographs*. Springer.

Goldschmidt, D., Wörgötter, F., and Manoonpong, P. (2014). Biologically-inspired adaptive obstacle negotiation behavior of hexapod robots. *Frontiers in Neurorobotics*, 8(3).

Kesper, P., Grinke, E., Hesse, F., Wörgötter, F., and Manoonpong, P. (2013). Obstacle/Gap Detection and Terrain Classification of Walking Robots based on a 2D Laser Range Finder. In *Proc. 16th Int. Conf. on Climbing and Walking Robots and the Support Technologies for Mobile Machines (CLAWAR)*, pages 419–426.

Manoonpong, P., Parlitz, U., and Wörgötter, F. (2013). Neural control and adaptive neural forward models for insect-like, energy-efficient, and adaptable locomotion of walking machines. *Frontiers in Neural Circuits*, 7(12).

Mittelstaedt, H. (1962). Control systems of orientation in insects. *Annual Review of Entomology*, 7(1):177–198.

Mittelstaedt, H. (1985). *Neurobiology of Arachnids*, chapter Analytical Cybernetics of Spider Navigation, pages 298–316. Springer Berlin Heidelberg, Berlin, Heidelberg.

Reynolds, A. M., Smith, A. D., Menzel, R., Greggers, U., Reynolds, D. R., and Riley, J. R. (2007). Displaced honey bees perform optimal scale-free search flights. *Ecology*, 88(8):1955–1961.

Triesch, J. (2005). *Artificial Neural Networks: Biological Inspirations – ICANN 2005: 15th International Conference, Warsaw, Poland, September 11-15, 2005. Proceedings, Part I*, chapter A Gradient Rule for the Plasticity of a Neuron's Intrinsic Excitability, pages 65–70. Springer Berlin Heidelberg, Berlin, Heidelberg.

Triesch, J. (2007). Synergies between intrinsic and synaptic plasticity mechanisms. *Neural Computation*, 19(4):885–909.

Vickerstaff, R. J. (2007). *Evolving dynamical system models of path integration*. PhD thesis, University of Sussex.

Vickerstaff, R. J. and Di Paolo, E. A. (2005). Evolving neural models of path integration. *Journal of Experimental Biology*, 208(17):3349–3366.

Wehner, R. and Srinivasan, M. V. (1981). Searching behaviour of desert ants, genus Cataglyphis (Formicidae, Hymenoptera). *Journal of comparative physiology*, 142(3):315–338.

Zenker, S., Aksoy, E., Goldschmidt, D., Wörgötter, F., and Manoonpong, P. (2013). Visual terrain classification for selecting energy efficient gaits of a hexapod robot. In *Advanced Intelligent Mechatronics (AIM), 2013 IEEE/ASME International Conference on*, pages 577–584.
